# Evaluating the genus *Cespitularia* MilneEdwards & Haime, 1850 with descriptions of new genera of the family Xeniidae (Octocorallia, Alcyonacea)

**DOI:** 10.3897/zookeys.754.23368

**Published:** 2018-05-02

**Authors:** Yehuda Benayahu, Leen P. van Ofwegen, Catherine S. McFadden

**Affiliations:** 1 School of Zoology, George S. Wise Faculty of Life Sciences, Tel Aviv University, Ramat Aviv, 69978, Israel; 2 Naturalis Biodiversity Center, P.O. Box 9517, 2300 RA Leiden, The Netherlands; 3 Department of Biology, Harvey Mudd College, Claremont, CA 91711, USA

**Keywords:** Indo-Pacific Ocean, new genera, phylogeny, sclerite microstructure, taxonomy

## Abstract

Several species of the family Xeniidae, previously assigned to the genus *Cespitularia* Milne Edwards & Haime, 1850 are revised. Based on the problematical identity and status of the type of this genus, it became apparent that the literature has introduced misperceptions concerning its diagnosis. A consequent examination of the type colonies of *Cespitularia coerulea* May, 1898 has led to the establishment of the new genus *Conglomeratusclera* **gen. n.** and similarly to the assignment of *Cespitularia simplex* Thomson & Dean, 1931 to the new genus, *Caementabunda* **gen. n.** Both new genera are described and depicted and both feature unique sclerite morphology, further highlighting the importance of sclerite microstructure for generic position among Xeniidae. Freshly collected material was subjected to molecular phylogenetic analysis, whose results substantiated the taxonomic assignment of the new genera, as well as the synonymies of several others.

## Introduction

Members of the octocoral family Xeniidae form a major faunistic component on shallow Indo-Pacific coral reefs (e.g., Alderslade 2001, Janes 2013, McFadden et al. 2014, Halàsz et al. 2014, 2015). They play a significant ecological role in coral reef ecosystems, exhibiting a rapid colonization rate (e.g., Tilot et al. 2008, Wild and Naumann 2013) as well as invasive capabilities (Ruiz-Allais et al. 2014). Uniquely among Octocorallia, in xeniids the pinnules along the margins of the polyp tentacles are commonly arranged in more than one longitudinal row. The number of pinnule rows and the number of pinnules in the outermost row have been considered taxonomically diagnostic (e.g., Hickson 1931a, Reinicke 1997, Halàsz et al. 2014). However, Halàsz et al. (2015) and subsequently McFadden et al. (2017) demonstrated that this character is not informative for species delineation among members of the xeniid genus *Ovabunda* Alderslade, 2001. The taxonomic literature on Xeniidae also considers several other morphological characters to be informative, such as colony shape, dimensions and coloration, as well as polyp retractability and pulsation in the live state (e.g., Reinicke 1997, Halàsz et al. 2014, 2015).

The majority of the described Xeniidae taxa have a high density of minute sclerites in their tissues, although some have only a few or none (e.g., Halàsz et al. 2014). This family has been considered to exhibit less diversity of sclerites than most other octocoral families, with the commonly held notion that most of the species feature relatively simple sclerites in the form of round platelets (Fabricius and Alderslade 2001). Consequently, most of the old taxonomic literature does not depict xeniid sclerites, although their size-range has occasionally been recorded (Halàsz et al. 2014 and references therein). Among the few studies that have included drawings of xeniid sclerites are those of *Cespitularia mantoni* Hickson, 1931, *C. multipinnata* Quoy & Gaimard, 1833 (in Hickson 1931: 168, fig. 5), and *C. stolonifera* Gohar, 1938 (in Utinomi 1950: 80, fig. 3f) (see also Halàsz et al. 2014). With the use of scanning electron microscopy (SEM), the diverse microstructural features of xeniid sclerites have now become evident (e.g., Benayahu 1990, 2010, Reinicke 1997, Alderslade 2001, Janes 2008, Aharonovich and Benayahu 2011, Halàsz et al. 2014). Subsequently, several new genera have been described, such as *Bayerxenia* Alderslade, 2001, *Ingotia* Alderslade, 2001, *Ixion* Alderslade, 2001, *Orangaslia* Alderslade, 2001, and *Yamazatum* Benayahu, 2010. Alderslade (2001) established the genus *Ovabunda* Alderslade, 2001 for previously described species of *Xenia* Lamarck, 1816 with the corpuscular sclerite-type, while retaining those with a dendritic surface in the original genus. To date, the phylogenetic studies on Xeniidae support the hypothesis that their distinct sclerite microstructure justifies establishing generic boundaries within this family (Haverkort-Yeh et al. 2013, McFadden et al. 2014).

There is considerable confusion in the literature concerning the diagnosis of the xeniid genus *Cespitularia*. This genus was erected by Valenciennes in an unpublished manuscript and later published by Milne Edwards and Haime (1850). The type of *Cornularia multipinnata* Quoy & Gaimard, 1833 collected in Tonga (West-Pacific) was designated to be the type species of the genus; Quoy and Gaimard (1833) also described *Cornularia subviridis* from the same locality. According to Milne Edwards and Haime (1850), the genus *Cespitularia* features non-retractile polyps arranged in fasciculi (=longitudinal groups) and united along the greatest part of their length by dense tough tissue; their description does not note the presence of sclerites. Our attempts to trace the types of both these *Cornularia* species have failed and therefore they are considered lost. Later, May (1899: 89) synonymized the genera *Cornularia* Quoy & Gaimard, 1833 and *Suensonia* Brundin, 1896 (see ahead) under *Cespitularia* Valenciennes; his description too does not note the presence of sclerites.

**Figure 1.**
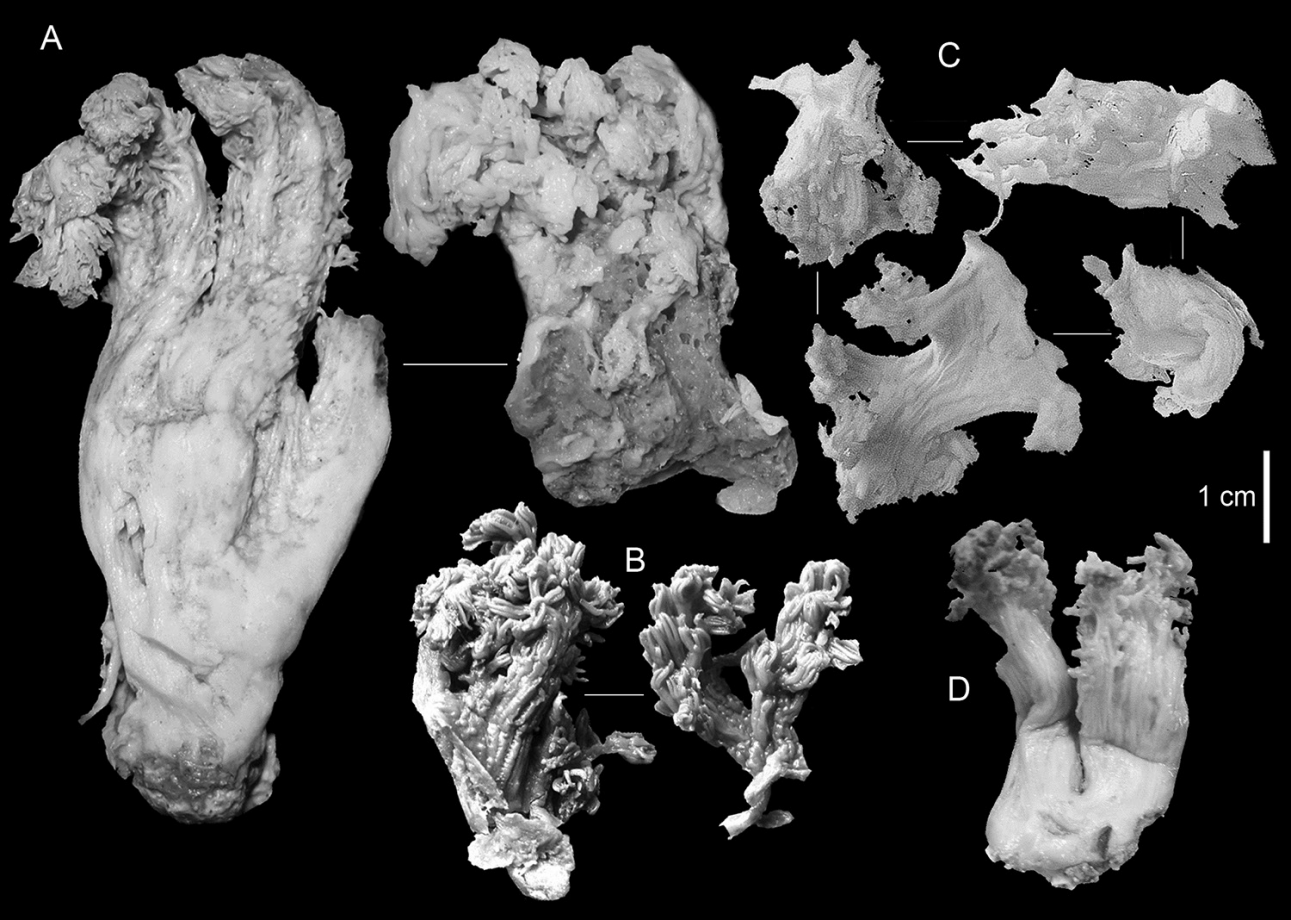
**A** *Conglomeratusclera coerulea* (May, 1898), syntypes ZMH C 2518 **B** *Conglomeratusclera coerulea* (May, 1898), type ZMB Cni 3671 **C** *Cespitularia taeniata* May, 1898, syntypes ZMH C 2519 **D** *Ammothea bauiana* May, 1898, type ZMH C 2375.

**Figure 2.**
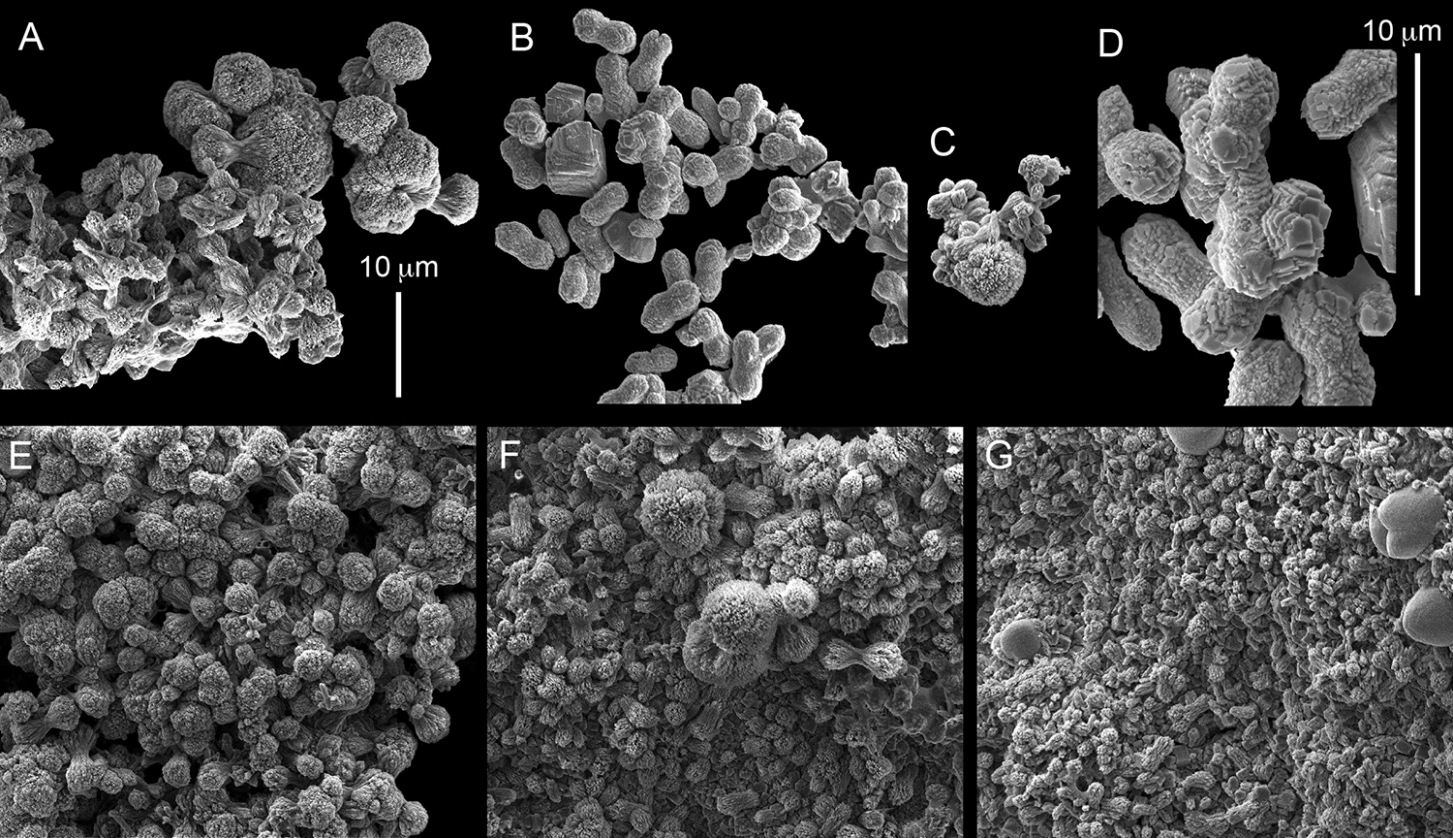
*Conglomeratusclera coerulea* (May, 1898), syntypes ZMH C 2518. **A** aggregate of spherules **B** conglomerate of dumbbells **C** conglomerate of spherules of various diameters **D** conglomerate of dumbbells **E–G** dense conglomerate of spherules with some dumbbells. Scale bar at **A** also applies to **B**, **C** and **E–G**.

**Figure 3.**
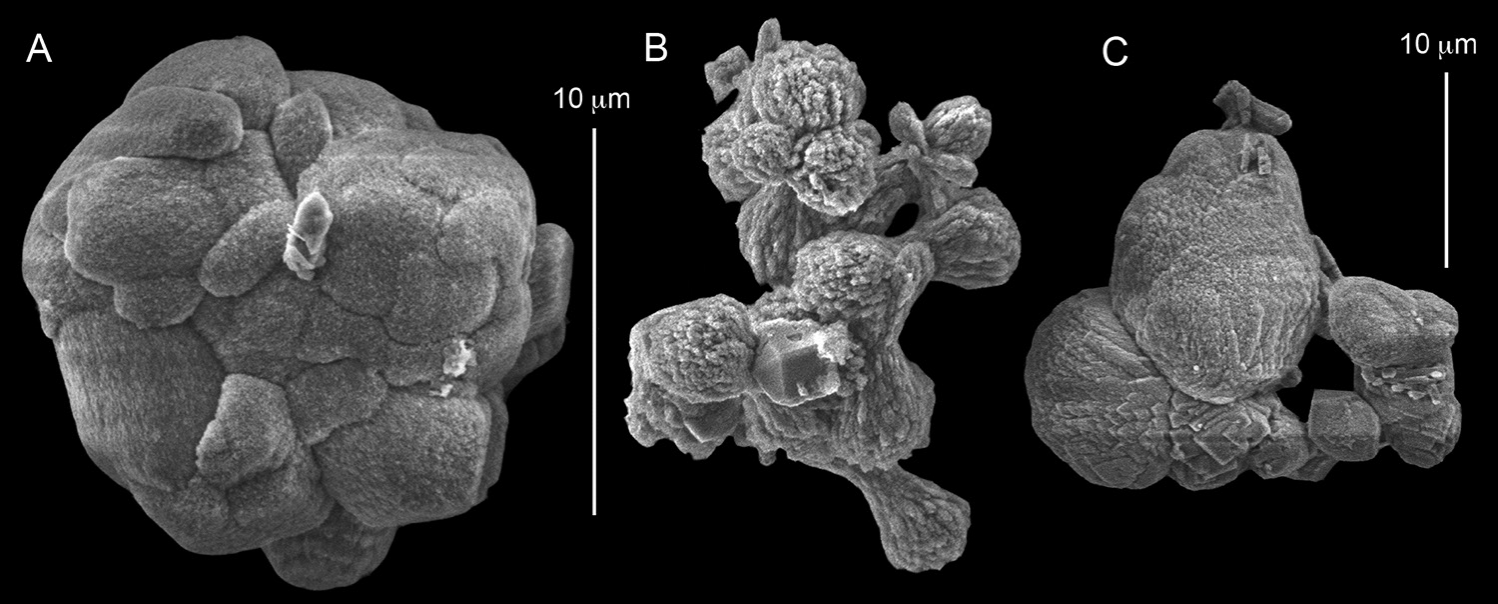
*Conglomeratusclera coerulea* (May, 1898), type ZMB Cni 3671. **A** conglomerate sclerites composed of spheres and spherules **B** conglomerate sclerite composed of spheres and dumbbells **C** conglomerate sclerite composed of spheres and spherules. Scale at **A** also applies to **B**.

**Figure 4.**
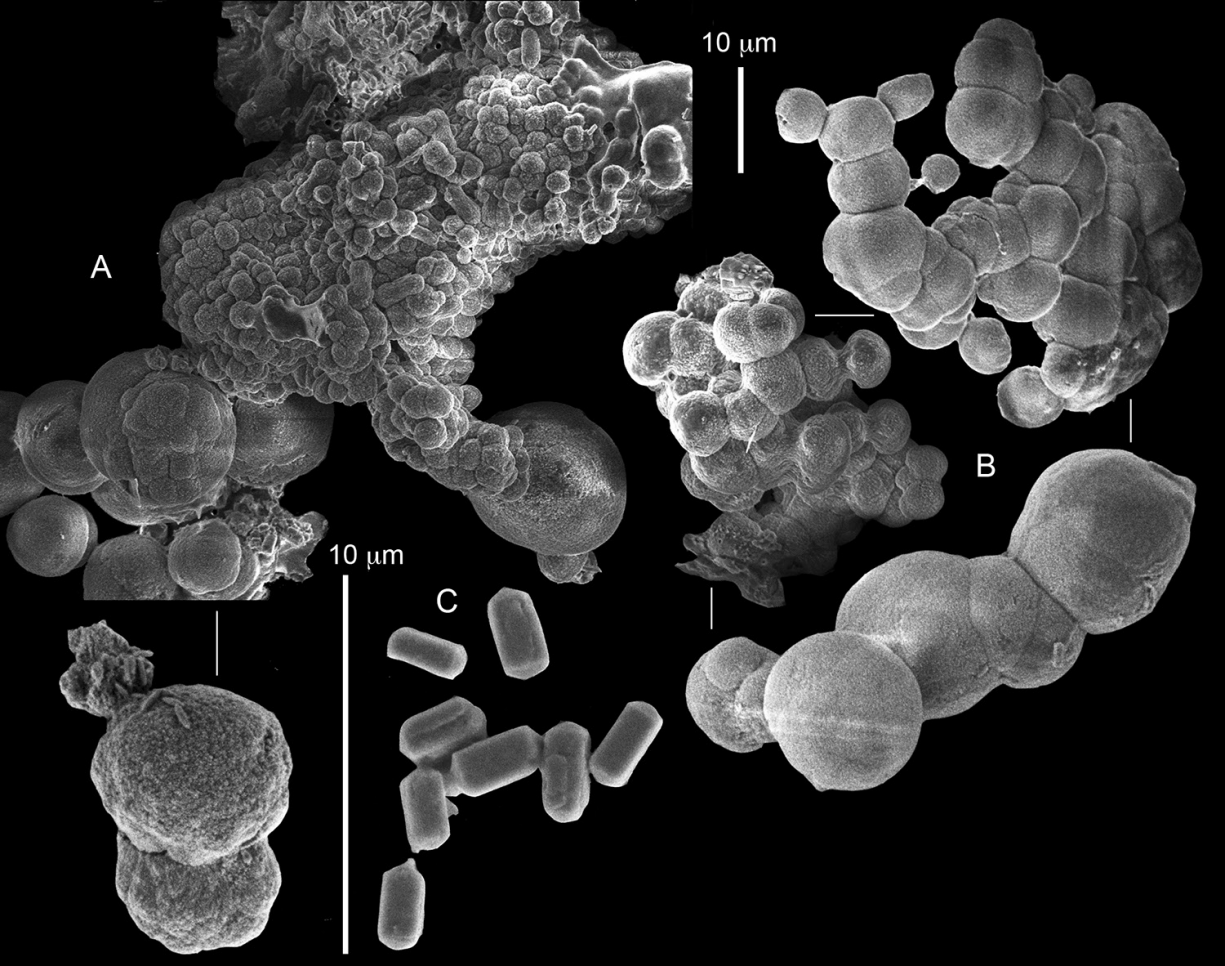
*Cespitularia taeniata* (May, 1898), type ZMH C 2519, synonym of *Conglomeratusclera coerulea* (May, 1898). **A** conglomerate sclerites composed of spherules and spheres **B** conglomerate sclerites composed of mainly spheres **C** cylinder-like small sclerites. Scale at **A** also applies to **B**.

**Figure 5.**
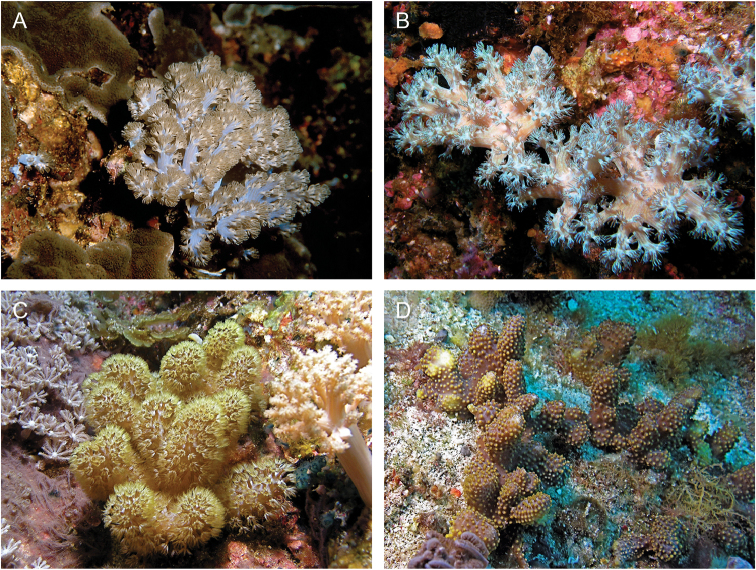
Live colonies on the reefs of Green Is. Taiwan. **A–B** *Conglomeratusclera coerulea* (May, 1898). **C** *Caementabunda simplex* (Thomson & Dean, 1931) with expanded polyps **D** *C. simplex* (Thomson & Dean, 1931) with partially retracted polyps. Photo credit Chang-Feng Dai, National Taiwan University, Taiwan.

**Figure 6.**
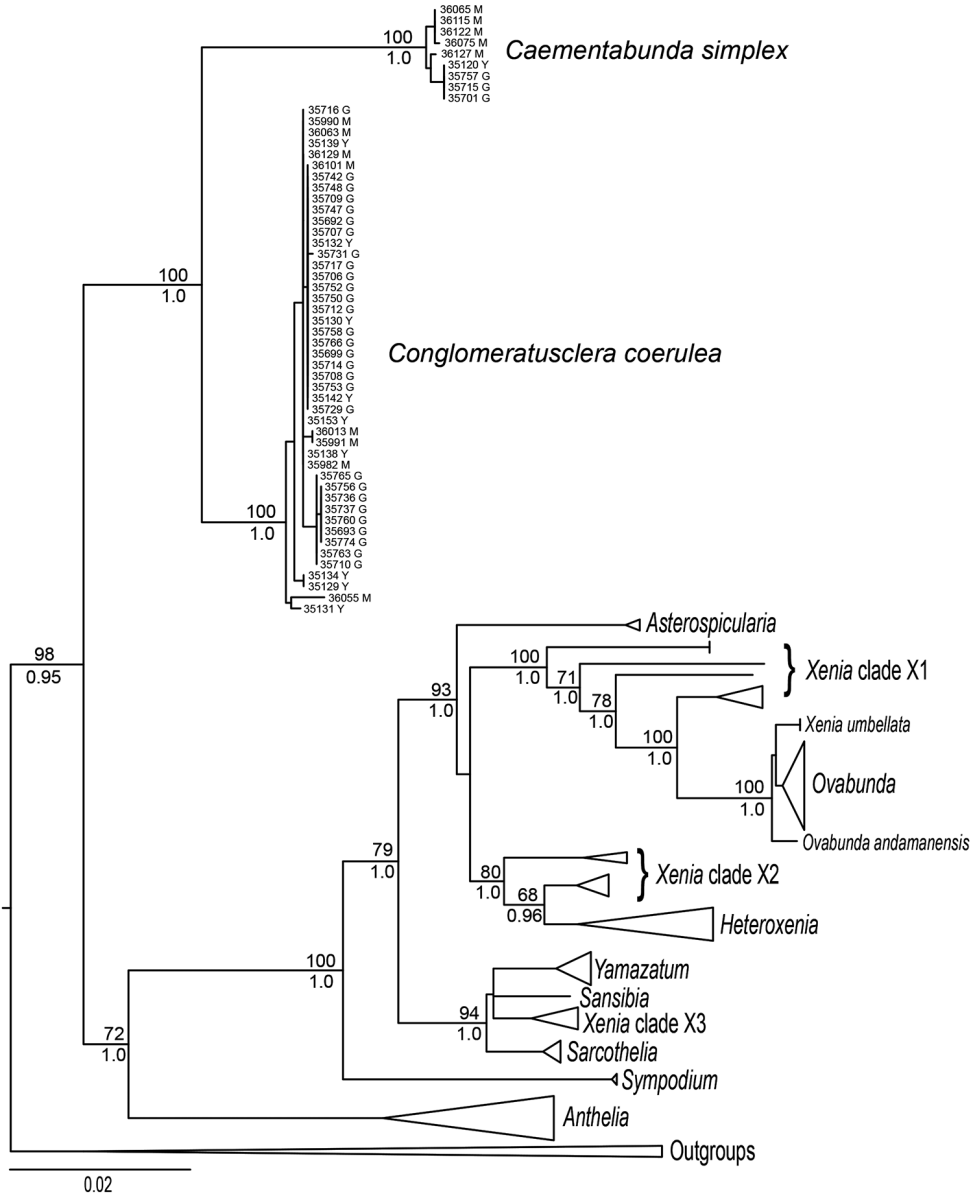
Maximum likelihood tree of family Xeniidae based on a partitioned analysis of concatenated *mtMutS*, *COI* and *28S* rDNA gene regions. Numbers above nodes: ML bootstrap percentages; numbers below nodes: Bayesian posterior probabilities. All genera and major clades of Xeniidae other than *Caementabunda* and *Conglomeratusclera* have been collapsed to facilitate readability. Specimens of *Caementabunda* and *Conglomeratusclera* are identified by ZMTAU catalog number and location of collection (G = Green Is., Taiwan; M = Madagascar; Y = Yonaguni Is., Japan).

**Figure 7.**
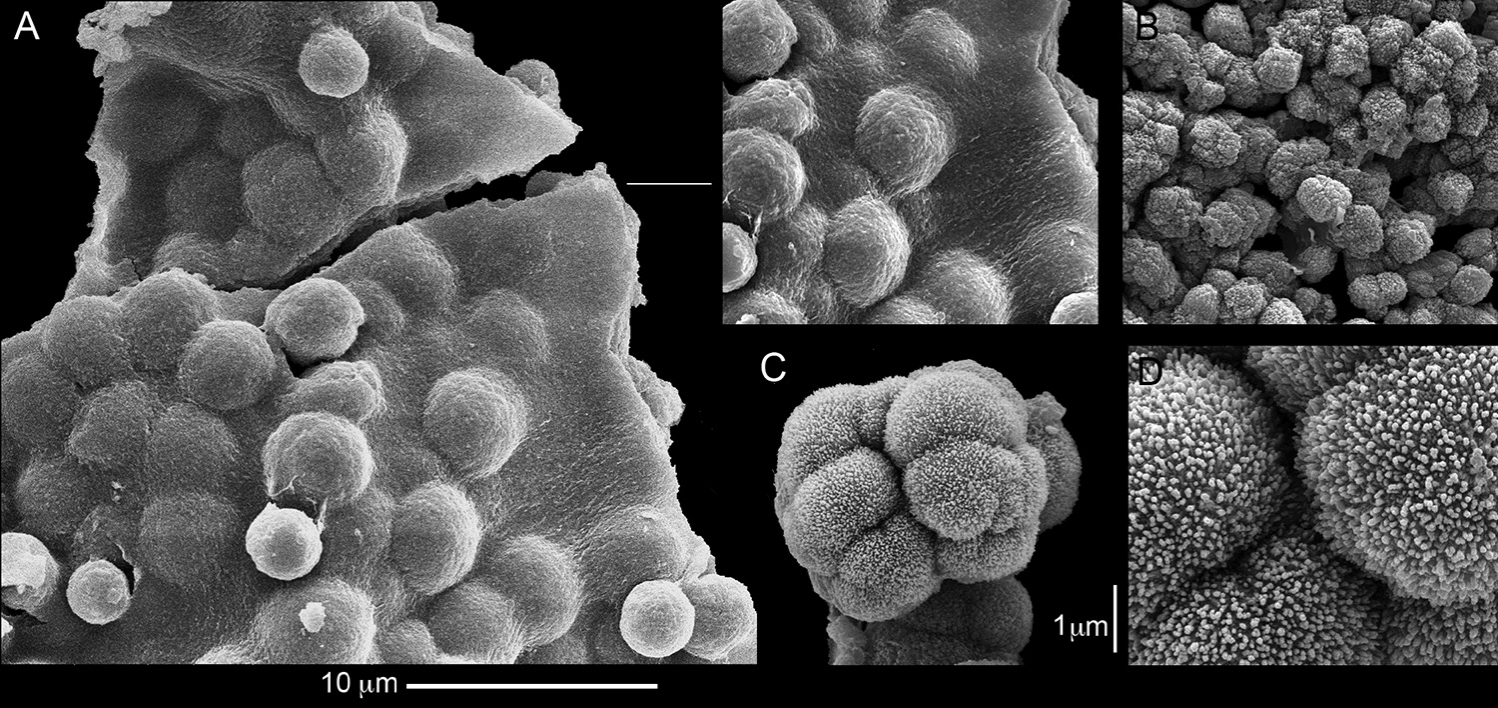
*Conglomeratusclera coerulea* (May, 1898), ZMTAU Co 35692. **A** spheres embedded in a calcareous lamella-like structure **B** conglomerate sclerite composed of spherules **C–D** spherules with bristly surface. Scale at **A** also applies to **B**, scale at **C** also applies to **D**.

Drawings of the type of *Cornularia multipinnata* Quoy & Gaimard (1833) (plate 22, figs 1–4), depict a colony with a distinct dome–shaped capitulum bearing polyps as well as a bare stalk with no polyps (fig. 4), thus resembling *Xenia* (see e.g., Fabricius and Alderslade 2001). *Cornularia subviridis* (plate 22, figs 5–7) was described as a colony with three elongated stems arising from a common base, each bearing polyps along half of its length (fig. 5). The depicted sclerites of this species are spindles (fig. 5’), but no information is given on those of *C. multipinnata*. Hickson’s (1931) revision of the Xeniidae (p. 162) referred to this original description, but erroneously stated that *C. multipinnata* featured spindle-shaped sclerites and *C. subviridis* had *Xenia*-like sclerites. Consequently, his conclusion that *C. subviridis* is probably *X. umbellata* would appear to be an error. Hickson’s revision also indicated that Quoy & Gaimard (1833) had provided errata, arguing that their original drawing of the two *Cornularia* species had been switched, and thus figs 1–4 should refer to *C. subviridis* and figs 5–7 to *C. multipinnata*. It should be noted that although the colony shape depicted in plate 22, fig. 5 might be considered to be *Cespitularia* (see Fabricius and Alderslade 2001), doubts nonetheless exist because of the spindle-shaped sclerites (fig. 5’). Such sclerites have never been recorded among Xeniidae and, therefore, doubt exists as to whether *Cornularia multipinnata* Quoy & Gaimard, 1833 should be assigned to the family Xeniidae. Until new xeniid material can be obtained from the original type locality (Tonga), the taxonomic status of *Cespitularia* *sensu stricto* Quoy & Gaimard (1833) cannot be unequivocally determined.

The genus *Cespitularia* Milne Edwards & Haime, 1850 was first revised by Kükenthal (1902), who diagnosed it as forming tree-like colonies, with polyps not positioned on a defined polypary. That revision listed the following species under the genus: *C. subviridis* (Quoy & Gaimard, 1833); *C. multipinnata* (Quoy & Gaimard, 1833) as well as the subsequently described species *C. mollis* (Brundin, 1896); *C. coerulea* May, 1898; and *C. taeniata* May, 1899. The revision by Hickson (1931) similarly diagnosed the genus as having dendritic branches with the margins of the capitulum not sharply defined, i.e., its polyps do not arise only from the summit of the branches but also from lower down, albeit gradually diminishing in number. Based on the problematical identity and status of the type of the genus *Cespitularia*, as detailed above, it is apparent that both of these revisions introduced further misperceptions concerning its diagnosis.

The ambiguity concerning the diagnosis of *Cespitularia* is further demonstrated in *C. mollis* (Brundin, 1896), originally described as *Suensonia mollis*, whose type locality is the Korean Straits (120 m depth). May (1899) assigned this species to *Cespitularia*, but Hickson (1931) stated that it “must be regarded as a distinct species” because of its geographical origin and depth of collection. Hickson (1931) also indicated that the sclerites of *S. mollis* are “twins, quadruplets and hour-glass shaped”, but no drawings were presented. Although Utinomi (1950:81) stated that this species is “a member of *Cespitularia*”, the type locality of *S. mollis* certainly departs from that of the tropical coral-reef systems. It can therefore be concluded that *C. mollis* is not a xeniid.

At present, the literature refers to 18 species of the genus *Cespitularia* (Cordeiro et al. 2018). Considering the fact that the type species of the genus *Cespitularia* is missing and presumed lost, we searched for the types of species that were originally assigned to the genus subsequent to the species noted above, i.e., from May (1898) onwards. Accordingly, the current study examined the following types: *C. coerulea* May, 1898, *C. taeniata* May, 1898, *C. simplex* Thomson & Dean, 1931, *C. robusta* Tixier-Durivault, 1966, and *C. turgida* Verseveldt, 1971. Freshly collected material was subjected to molecular phylogenetic analyses whose results also substantiated the taxonomic findings that have led us to assign new xeniid genera as well as to synonymize several others. Examination of diverse, related museum material provided data on intraspecific variation and the zoogeographical distribution of the taxa.

## Materials and methods

The study examined preserved type specimens obtained on loan from the British Museum of Natural History (**BMNH**); Muséum National d’Histoire Naturelle, Paris (**MNHN**); Naturalis Biodiversity Center, formerly Rijksmuseum van Natuurlijke Historie, Leiden (**RMNH**); Zoologisches Museum, Hamburg (**ZMH**); Zoologisches Museum Berlin (**ZMB**); Smithsonian National Museum of Natural History, Washington DC (**USNM**), and the Steinhardt Museum of Natural History at Tel Aviv University (**ZMTAU**).

Morphological features of the preserved colonies were recorded, comprising dimensions, branching and stalk length, and width of the stalk at the colony base. The number of rows of pinnules and number of pinnules on the aboral side of the tentacles were counted under a dissecting microscope, whenever possible from multiple polyps. The length of the anthocodiae, consisting of the polyp body and extended tentacles, and the dimensions and shape of the pinnules were also recorded (see also Halàsz et al. 2014). To examine the sclerites, the tissue samples were treated with 10 % sodium hypochlorite followed by repeated rinses in distilled water. Wet preparations of the clean sclerites from polyps and the colony base were examined under a Nikon Optiphot light microscope at 400× magnification. Observed differences led to preparation of SEM mounts from both regions; otherwise only those from the polyp were used (see Aharonovich and Benayahu 2011); each stub usually contained numerous sclerites and samples were coated with Pd/Au and viewed under a Quanta 200 FEG (Field Emission Gun) ESEM at 5–20 kV. SEM was used to examine sclerites of almost all the studied material; in certain cases, wet preparations were prepared and examined under the light microscope (×200–400). Both SEM and wet preparations were prepared from the same colonies in order to correctly visualize the unique structure of the corresponding sclerites. The zoogeographical species distributions were determined by the examination of types and other material.

Freshly collected material used for molecular and morphological studies was collected by YB in Yonaguni Is., Ryukyu Archipelago, Japan (in 2010); Green Is., Taiwan (2012) and Nosy Be, Madagascar (2015). Xeniidae colonies tend to release large quantities of mucus, especially when being detached from the reef and brought onboard, which is particularly relevant to the taxa studied here. This usually causes rapid colony disintegration and poor condition of museum material. Therefore, upon collection samples were immediately preserved in 95 % ethanol and subsamples were removed and preserved in absolute ethanol for molecular studies and then placed on ice in cool boxes until brought to the laboratory. In order to ensure appropriate preservation, the fixatives were replaced twice within 24 hours after collection, and throughout all preservation steps, the bottles were shaken to enhance infiltration of the fixative into the tissues.

DNA was extracted from ethanol-preserved tissue samples using the Qiagen DNEasy Blood & Tissue kit, and three gene regions were subsequently amplified by polymerase chain reaction (PCR) using previously published primers and PCR protocols (McFadden et al. 2011). For most specimens, we amplified the octocoral-specific mitochondrial *mutS* homolog (*mtMutS*) using primers ND42625F (McFadden et al. 2006) and mut3458R (Sánchez et al. 2003); cytochrome oxidase I (*COI*) and the adjacent intergenic region, *igr1*, using primers COII8068F (McFadden et al. 2004) and HC02198 (Folmer et al. 1994); and a fragment of 28S rDNA using primers 28S-Far and 28S-Rar (McFadden and Ofwegen 2013). The L-INS-i method in MAFFT (Katoh et al. 2005) was used to align sequences to a reference dataset consisting of previously published sequences for other genera of xeniids and three outgroup taxa, *Coelogorgia*, *Paralemnalia*, and *Rhytisma* (McFadden et al. 2014). Pairwise measures of genetic distance (Kimura 2-parameter) among sequences were computed using MEGA v.5 (Tamura et al. 2011). jModelTest2 (Darriba et al. 2012) was used to select appropriate models of evolution for maximum likelihood analyses that were run using GARLI 2.0 (Zwickl 2006). Analyses were run for each gene region alone, and for a combined dataset with all three genes concatenated. Each gene was treated as a separate data partition with different models of evolution applied to each (*mtMutS*: HKY+G; *COI*: TIM2+I+G; *28S*: GTR+I+G). Bayesian analyses of the concatenated alignment used MrBayes v. 3.2.1 (Ronquist et al. 2012) with the same data partitions and evolutionary models applied, except that GTR+I+G was substituted for TIM2+I+G. MrBayes was run for 5,000,000 generations (until standard deviation of split partitions < 0.01) with a burn-in of 25 % and default Metropolis coupling parameters.

## Systematics

### Class Anthozoa Ehrenberg, 1831

#### Subclass Octocorallia Haeckel, 1866

##### Order Alcyonacea Lamouroux, 1812

###### Family Xeniidae Ehrenberg, 1828

####### Conglomeratusclera gen. n.

Taxon classificationAnimaliaAlcyonaceaXeniidae

http://zoobank.org/F3E23C0E-B3D8-4C72-9638-33404B685A1B

######## Type species.

*Cespitularia coerulea* May, 1898: 21

######## Diagnosis.

Colonies soft with a short but distinct stalk, ramified into primary branches and occasionally into secondary ones. Polyps monomorphic, found along the branches, sometimes down on the stalk; most are non-retractile. Sclerites of a wide diversity of forms and dimensions, many lacking a distinct repetitive morphology. They include spheres, spherules, and small dumbbell-like sclerites. They are commonly cemented together, forming heterogeneous morphologies of various shapes and sizes. Occasionally, the aggregates form plate-like structures, embedded with spheres and/or spherules. The abundance of sclerites can vary greatly; in some specimens they are rare and then mostly found only at the colony base, and occasionally they may be found in all parts of the colonies, or may even be entirely absent. Zooxanthellate.

######## Etymology.

The generic name is derived from Latin *conglomerātus*, which refers to anything composed of heterogeneous materials or elements and *sclera* from Greek meaning sclerite. Here it denotes the sclerites that resemble the geological structures termed conglomerates, a feature comprising rounded to sub-angular clast of granules, pebbles or cobbles cemented together. Gender female.

####### Conglomeratusclera coerulea

Taxon classificationAnimaliaAlcyonaceaXeniidae

(May, 1898)

Figures 1A–B , 2 , 3 , 4 , 5A–B , 7 , 8 , 9 , 10 , 11 , 12 , 13 , 14 , 15 , 16 , 17 , 18 , 19 , 20 , 21 , 22 , 23 , 24 , 25

Cespitularia coerulea May, 1898: 21; May 1899: 90, plate I, fig. 10; Kükenthal 1902: 659; Thomson and Henderson 1906: 414–415; Thomson and Mackinnon 1910: 173, plate 12, fig. 5; Hickson 1931: 162 (listed only); Thomson and Dean 1931: 32–33; Roxas 1933: 106, plate 4, fig. 6; Malyutin 1992: 2 (listed only); Benayahu et al. 2004: 551 (listed only).
Cespitularia taeniata May, 1899: 89–90; Kükenthal 1902: 659, Hickson 1931: 162; Utinomi 1950: 14–15, fig 3b, c; 1954: 102 (listed only); Thomson and Mackinnon 1910: 172, Thomson and Dean 1931: 33.

######## Material.

**Syntypes: ZANZIBAR**: ZMH C 2518, Kokotoni, two colonies and two fragments, Tumbatu (southern reef), 24 July 1885, coll. Stuhlmann; ZMB Cni 3671, two colonies, 1885, coll. Sander; **types** of *Cespitularia taeniata*; **MOZAMBIQUE**: ZMH C 2519, three colonies and three fragments, coll. Philippi, 1884.

######## Other material.

**JAPAN**: ZMTAU Co 29285, Yonaguni Is., Ryukyu Archipelago, coll. Y. Benayahu, 13 November 1992, ten specimens; ZMTAU Co 29290, Nurugan, Yonaguni Is., Ryukyu Archipelago, 04°05'N, 122°57'E, 23 m depth, coll. Y. Benayahu, 11 November 1992, ZMTAU Co 31699, details as before, six specimens; ZMTAU CO 35129, West Point, Yonaguni Is., Ryukyu Archipelago, 11–22 m depth, coll. Y. Benayahu, 4 July 2010, two specimens; ZMTAU CO 35130, details as before; ZMTAU Co 35131, details as before, four specimens ZMTAU Co 35132, Co 35134, Co 35138, Co 35139, details as before; ZMTAU Co 35142, West Point, Yonaguni Is., Ryukyu Archipelago, 16–22 m depth, coll. Y. Benayahu, 5 July 2010, two specimens; ZMTAU Co 35153, details as before; **KENYA**: ZMTAU Co 31326, Nyali, off Mombasa, 10–16 m depth, coll. Y. Benayahu & S. Perkol, 1 February 2001; ZMTAU Co 31635, Turning Bouya, Shelly Reef, off Likoni, 04°05'S, 39°41.1'E, 15–28 m depth, coll. Y. Benayahu, 27 February 2002, two specimens; **MADAGASCAR**: ZMTAU Co 35982, Riva Be, 12°59.126'S, 48°34.453'E, 8–10 m depth, coll. Y. Benayahu, 27 November 2012, three specimens; ZMTAU Co 35990, Riva Be, 12°59.094'S, 48°34.622'E, 10–11 m depth, coll. Y. Benayahu, 27 November 2012, two specimens; ZMTAU Co 35991, details as before, four specimens; ZMTAU Co 36013, Ankaréa, 12°50.054'S, 48°34.563'E, 6–9 m depth, coll. Y. Benayahu, 29 November 2012; ZMTAU Co 36055, Co 36063, 4 Fréres, 12°59.655'S, 48°29.248'E, 4–15 m depth, coll. Y. Benayahu, 1 December 2012; ZMTAU Co 36101, Ronald Point, Nosy Be, 13°23.530'S, 48°00.143'E, 19–27 m depth, coll. Y. Benayahu, 3 December 2012, two specimens; ZMTAU Co 36129, Ronald Point, Nosy Be, 13°29.032'S, 47°58.721'E, 2–14 m depth, coll. Y. Benayahu, 3 December 2012, two specimens; USNM 54000 Nosy Be; USNM 54003 Nosy Be; **MOZAMBIQUE**: ZMTAU Co 31296, Ilha Sete Paus, 14°58.572'S, 40°47.389'E, 6 m depth, coll. M. Schleyer, 16 November 2000, two specimens; ZMTAU Co 31337, Ilha Caldeira, 16°38'22"S, 39°43'10"E, 4–16 m depth, coll. M. Schleyer, 2 June 2000, four specimens; **TAIWAN**: ZMTAU, Co 32988, Lomenyan, Green Is., 22°40'56"N, 121°30'06"E, 3–25 m depth, coll. Y. Benayahu, 12 July 2005; ZMTAU Co 33006, details as before, seven specimens, Co 33008, details as before; ZMTAU Co 33030, Dabaisha, Green Is., 22°38'25"N, 121°29'04"E, 10–25 m depth, coll. Y. Benayahu, 14 July 2005; ZMTAU Co 33036, Co 33043, 33045, Nanliao, Green Is., 22°39'40"N, 121°27'59"E, 10–25 m depth, coll. Y. Benayahu, 14 July 2005; ZMTAU Co 35693, Co 35699, Co 35708, Co 35709, Co 35712, Co 35714, Co 35716 , Co 35717, (only molecular sample), Shihlang, Green Is., 22°39.425'N, 121°28.399'E, 8–12 m depth, coll. Y. Benayahu, 3 September 2012, ZMTAU Co 35692, details as before, three specimens; ZMTAU Co 35706, Co 35707, details as before, two specimens; ZMTAU Co 35725, Dabaisha, Green Is., 22°38.284'N, 121°29.457'E, 14–25 m depth, coll. Y. Benayahu, 4 September 2012; ZMTAU Co 35729, details as before, two specimens; ZMTAU Co 35731, details as before, three specimens; ZMTAU Co 35736, Co 35737, Dabaisha, Green Is., 22°38.284'N, 121°29.457'E, 11–15 m depth, coll. Y. Benayahu, 4 September 2012; ZMTAU Co 35742, details as before, two specimens; ZMTAU Co 35747, Co 35748, Co 35750, Co 35753, Iron Artificial Reef, Green Is., 22°38'33"N, 121°28'31"E, 20–26 m depth, coll. Y. Benayahu, 5 September 2012; ZMTAU Co 35752, details as before, three specimens, ZMTAU Co 35756, Co 35758, Co 35760, Co 35763, Co 35765, Co 35774, Shihlang, Green Is., 22°39.425'N, 121°28.399'E, 7–10 m depth, coll. Y. Benayahu, 5 September 2012; ZMTAU Co 35759, details as before, two specimens; ZMTAU Co 36232, Co 36235, Shihlang, Green Is., 22°39'17.91"N, 121°28'26.41"E, 6–11 m depth, coll. Y. Benayahu, 26 August 2013; ZMTAU Co 36247, details as before, four specimens; ZMTAU Co 36255, Gueiwan, Green Is., 22°38'41"N, 121°28'26"E, 10–18 m depth, coll. Y. Benayahu, 27 August 2013, two specimens; **MAYOTTE**: ZMTAU Co 37403, Glorioso Is., 11°34.880'S, 47°16.862'E, 10–11.5 m depth, coll. M. Schleyer, 20 November 2016, two specimens; ZMTAU Co 37430, Saziley, 12°59.138'S, 45°10.947'E, 3–4 m depth, coll. M. Schleyer, 26 June 2011; ZMTAU Co 37431, Station East Bouzi, 12°48.739'S, 45°14.543'E, 5–10 m depth, coll. M. Schleyer, 24 June 2011; **MAURITIUS**: BMNH 1912.2.24.65; BMNH 1912.2.24.66; Cargados Carajos, 20–25 m depth; BMNH 1933.3.13.175, Cargados Carajos, 20–25 m depth, coll. J.A. Thomson; BMNH 1933.3.13.176, Cargados Carajos, 20–30 m depth, Percy Sladen Trust Expedition, coll. J.A. Thomson; BMNH 1933.5.3.301, Port East Africa, Sir J.A. Thomson Expedition, 11 November 1907; **MALAYSIA**: BMNH 1985.4.17.20, NE Borneo, Sabah, Semporna, Pulau-Pulau Mantanani. **AUSTRALIA**: USNM 60795, Great Barrier Reef, Myrmidon Reef, Northern Reef, 17°00'S, 146°00'E Queensland, 1982; **INDONESIA**: RMNH Coel 42158, SW Sulawesi, Spermonde Archipelago, west of Lumu-Lumu Is.; RMNH Coel 42159, N Sulawesi, Bunaken park, ESE Siladen Is.; RMNH Coel 42161, Snellius II Exp. Station 4.139, NE Taka Bone Rate (Tiger Is.), S. of Tarupa Kecil, edge of reef flat, 06°30'S, 121°08'E, SCUBA, snorkeling on sea grass bed, 30 m depth, 25–26 September, 13 and 17 October 1984; RMNH Coel 42162, N. Sulawesi, Selat Lembeh, Pulau Lembeh, N of Pulau Burung, 01°29'N, 125°15'E; sandy bay merging to the north in stony boulders beach, stony and soft corals, SCUBA, 22 October 1994, 2–25 m depth, coll. L.P. van Ofwegen; RMNH Coel 42163 N. Sulawesi, Selat Lembeh, Pulau Lembeh, Air Bajo, near Kereko, Nusu Dua; SUL 13, 01°29'N, 125°15'E; sandy bay between rocks, N-exposed, gently sloping bottom with large boulders, snorkeling 5 m depth, 21 October 1994, coll. J.C. Den Hartog; RMNH Coel 42165, Buginesia Prog. UNHAS-NNM, SW Sulawesi. Spermonde Archipelago N of Kudingareg Keke (=14 km WNW of Makassar), 5°06'S, 119°17'E, SCUBA, 5–25 m depth, 1994 Sul. KK SW, 14 October 1994, coll. B.W. Hoeksema; RMNH Coel 42166, Buginesia Prog. UNHAS-NNM, SW Sulawesi, Spermonde Archipelago N of Langkai Is. (=37 km WNW of Makassar), 5°02'S, 119°05'E, coral reef, SCUBA, 24 June 1994, coll. B.W. Hoeksema; RMNH Coel 42167, Buginesia Prog. UNHAS-NNM, SW Sulawesi, Spermonde Archipelago N of Langkai Is. (=37 km WNW of Makassar), 5°02'S, 119°05'E, coral reef, SCUBA, 24 June 1994, coll. B.W. Hoeksema; RMNH Coel 42170, Buginesia Prog. UNHAS-NNM, SW Sulawesi, Spermonde Archipelago, N of Kudingareng Keke (=14 km WNW of Makassar), 5°0'S, 119°17'E, SCUBA, 1994 Sul. KK SW, 5 September 1994, coll. B.W. Hoeksema; **PHILIPPINES**: RMNH Coel 42160, Cebu strait Expedition, Station CEB. 13.

######## Notes to previous description.

The original description of *C. coerulea* by May (1898) referred to a colony from Kokotoni, Zanzibar. Later, May (1899) repeated the description, referring to colonies collected from that location in 1889 by Stuhlmann and from Zanzibar in 1885 by Sander, deposited in Hamburg and Berlin museums, respectively. During a visit by the senior author to ZMH two colonies were found labeled as the type of *C. coerulea*, both collected in Kokotoni, Zanzibar, 24 July 1895 (leg. Stuhlman). Similarly, in a subsequent visit to ZMB two colonies were found, labeled as syntypes of *C. coerulea*, collected in Zanzibar, 1895 (leg. Sander). Both ZMH and ZMB colonies are considered to be the original syntypes of that species and are re-described below.

######## Description.

ZMH C 2518 consists of two colonies; the first is 8.5 cm high by 4.2 cm wide and the second 5 cm high by 4 cm wide (Figure 1A). The polypary of these colonies is branched and their tips are bent. They bear non-retractile polyps, with some occurring towards the upper part of the colony’s base. The polyp body is up to 8 mm long and the tentacles are up to 3 mm long; the latter bear one row of pinnules and 16–18 pinnules along each edge. The pinnules are short, pointed and evenly placed along the tentacle, with a narrow space of less than a pinnule width between adjacent ones. The preserved colonies are pale gray- almost white. Sclerites could not be found in the upper part of the branches or in the polyps. However, the lower part of the branches, including the base of the colonies, feature conglomerates, comprised of spherules and small dumbbell-like sclerites, mostly cemented (Figure 2). The spherules are about 0.002–0.006 mm in diameter (Figures 2A, E–G), with a rather rough surface-texture. The abundance of the dumbbells (Figures 2B–D, F) may exceed that of the spheroids. The former vary in size, with a length of 0.003–0.006 mm. The conglomerate nature of the sclerites exhibits a large morphological variation as demonstrated in Figure 2. The syntype ZMB Cni 3671 (Figure 1B) resembles syntype ZMH 2518, except for the size of the colonies. Most of the polyps of the former are expanded, well-preserved, and thus recognizable on the branches of the colony. The sclerites are similar, conglomerated spheres and spherules along with some double-heads (Figure 3), but are less common in the tissues compared to ZMH C 2518. Under the light microscope wet preparations of the tentacles removed from ZMB Cni 3671 revealed some conglomerates along with spheres of various sizes.

The type material of *Cespitularia taeniata* (ZMH C 2519) comprises two flaccid colonies and two additional fragments (Figure 1C). The colonies are 3–4.5 cm high by 2–2.5 cm wide. Their polyparies consist of short branches bearing non-retractile polyps; some polyps were also found on the upper part of the stalk. The tentacles feature one row of 16–18 pointed pinnules, evenly placed along the edges with a free space between adjacent ones. Sclerites were found in the base of the colonies and the branches (Figure 4) but none in the polyps. They are conglomerates comprised mainly of spherules (Figure 4A) and some predominantly of spheres (Figure 4B), the latter measuring up to 0.018 mm in diameter. In addition, some cylinder-like small sclerites featuring round tips are also found, measuring 0.002–0.003 mm (Figure 4C). It should be noted that the aggregates tend to disintegrate during the sclerite preparation and therefore their actual dimensions cannot be determined.

A colony labeled as ZMH C 2375 (Figure 1D) features tentacles with 12–14 pinnules and sclerites similar to ZMH C 2519. ZMH C 2375 is listed in the museum’s catalog as the “Typus von *Ammothea bauiana* May, 1898” along with a note that Gohar had corrected the identification in 1938 to *C. taeniata*. Both colonies, ZMH C 2519 and ZMH C 2375, are light gray-beige. *Conglomeratusclera taeniata* was described by Thomson and Dean (1931: 33) as being “near to but distinct from *Cespitularia coerulea*”. The current findings indicate that there are only some small morphological differences in the colony and polyp dimensions between the two species, and therefore, the above statement appears reasonable. Utinomi (1950) described the *C. taeniata* specimen identified by him as having 10–12 pinnules, slightly lower in range compared to the 12–14 pinnules of C 2375. The current examination of the types of both *C. coerulea* and *C. taeniata* revealed that despite the erroneous statement that they have no sclerites, they feature quite similar sclerites. It is therefore suggested that the similarity between the two species indicates that the above-reported morphological differences in the number of pinnules of the two types represent intra-specific variation. The sequencing results obtained in the current study along with the morphological findings further substantiate this conclusion, as colonies with a single row of 8–22 pinnules share similar DNA sequences (see ahead). Therefore, it is concluded that *C. coerulea* and *C. taeniata* *sensu stricto* should be synonymized, and both are now designated under *Conglomeratusclera coerulea*.

######## Remarks.

The original descriptions of *Cespitularia coerulea* by May (1898, 1899) indicated an absence of sclerites in the colony. In contrast, the current findings demonstrate the presence in the syntypes of a novel type of sclerite, depicted here for the first time. These sclerites are composed of agglomerated calcite-constructed minute substructures of various morphologies, mostly spherules, spheres, and double heads appearing in different arrangements. They were probably overlooked in previous studies due to their minute size and also occasional low abundance. Moreover, the unusual irregular sclerite morphology with almost no definite structure (Figures 2–4), may have caused the misinterpretation concerning their potential as octocoral sclerites to be used as diagnostic characters for taxonomic purposes.

Since the original description of *C. coerulea* a number of studies have assigned specimens to that species. Thomson and Henderson (1906) identified a multi-branched colony from Zanzibar, with one row of pinnules and no sclerites. Later, Thomson and Mackinnon (1910) described a similar colony from Cargados Carajos (Mauritius), noting that when alive the colony was “vivid grass green, but after preservation it faded to cream”, a feature that has been widely observed in the current study (see below). Thomson and Dean (1931) identified *C. coerulea* from Kawassang, Indonesia, obtained in the course of the Siboga Expedition, featuring a single row of pinnules and no sclerites, with no mention of the number of pinnules in the polyps. Next, Roxas (1933) identified the same species from Sabang, near Puerto Galera, Mindoro, Philippines, with one row of 14–18 pinnules and no sclerites. Interestingly, that study of Roxas’s study was the first to indicate number of the pinnules in that species. In general, the above octocoral samples are in agreement with the original description by May (1898), but all the above authors nonetheless failed to detect any sclerites.

######## Color.

When alive, the color of colonies ranges from vibrantly bluish-purple, light green, light yellow-beige, light cream to almost white (see Figure 5A, B). The alcohol-preserved colonies lose their vibrant colors and mostly become pale cream, gray, or beige.

######## Morphological variation.

In the current study, examination of the colonies from Green Is., Yonaguni Is. and Madagascar was based on both morphological characters (colony shape, pinnule count, and sclerite features), along with DNA sequencing; the latter enabled us to construct a phylogenetic tree (Figure 6). In general, the colony shape of all the colonies listed in Material Examined was in agreement with the syntypes shown above, except for colony size. All colonies exhibited one row of pinnules along the margins of the polyp tentacles, with a variable number of pinnules, ranging from 8 to 22 per row. In some colonies the tentacles were partially or completely withdrawn or the pinnules fully contracted, probably due to the preservation process. In several cases the polyps were fully expanded and in others partially or fully contracted.

The following findings denote the number of pinnules found in some of the sequenced colonies (Figure 6), demonstrating the variability in pinnule count. The respective colonies from Green Island are ZMTAU Co 35717: 8, Co 35747: 8, Co 35774: 8, Co 35742: 8–9, Co 35750: 8–9, Co 35753: 8–9, Co 35714: 10–11, Co 35712: 11–12, Co 33045: 11–16, Co 35692: 11–16, Co 35707: 11–16, Co 35699: 12–15, Co 35709: 15, Co 35758: 15, Co 35693: 15–16, Co 35729: 15–18, Co 35693: 16, Co 35725: 16–17, Co 35748: 16–18, Co 35763: 18–20, Co 35756: 20, Co 35760: 20, Co 35736: 21–22 and Co 35737: 21–22; colonies with fully contracted pinnules Co 35706, Co 35708, Co 35710, Co 35731, Co 35752, Co 35765, and Co 35766. Colonies from Yonaguni Is are ZMTAU Co 35131: 9–12 pinnules, Co 35132: 12–14 and Co 35134: 11–13. Colonies from Madagascar: ZMTAU Co 36013: 10–13 pinnules and Co 36129: 12–13.

**Figure 8.**
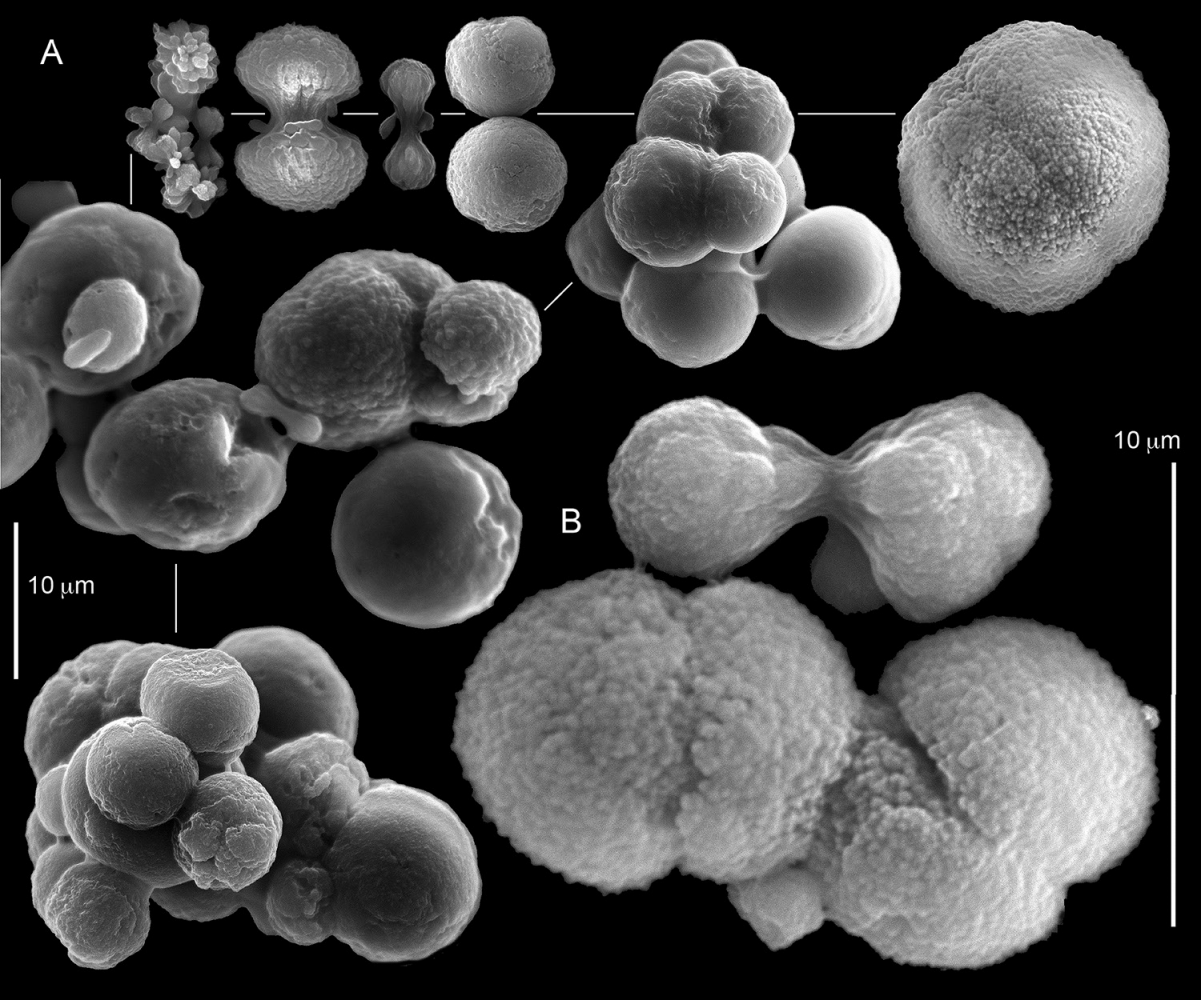
*Conglomeratusclera coerulea* (May, 1898), ZMTAU Co 35737. **A** conglomerate sclerite composed of spherules and of spheres, dumbbells **B** bristly surface of dumbbells and double spheres.

**Figure 9.**
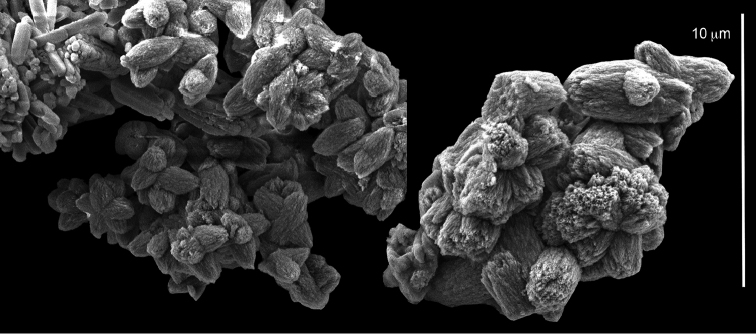
*Conglomeratusclera coerulea* (May, 1898), ZMTAU Co 35765. Conglomerate sclerite composed of striated ovals and cylinder-like small sclerites (left top corner).

**Figure 10.**
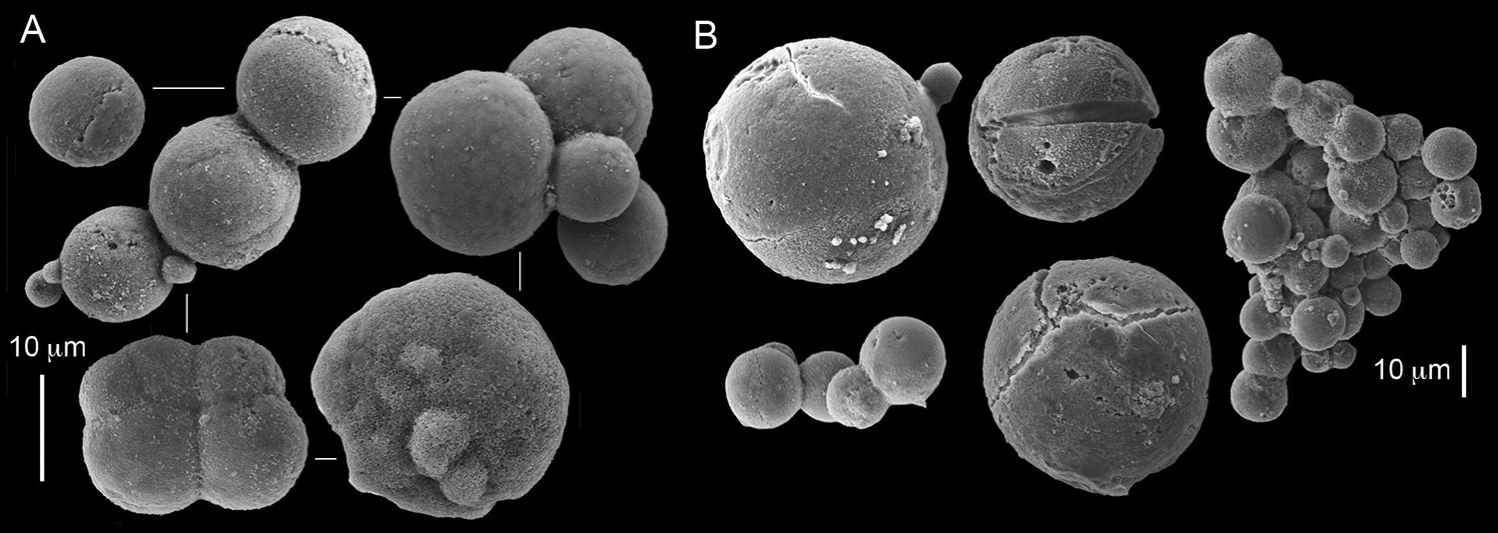
*Conglomeratusclera coerulea* (May, 1898), ZMTAU Co 35709. **A** individual sphere, conglomerate sclerites composed of spheres and spherules **B** individual spheres, conglomerate sclerites composed of spheres and spherules.

**Figure 11.**
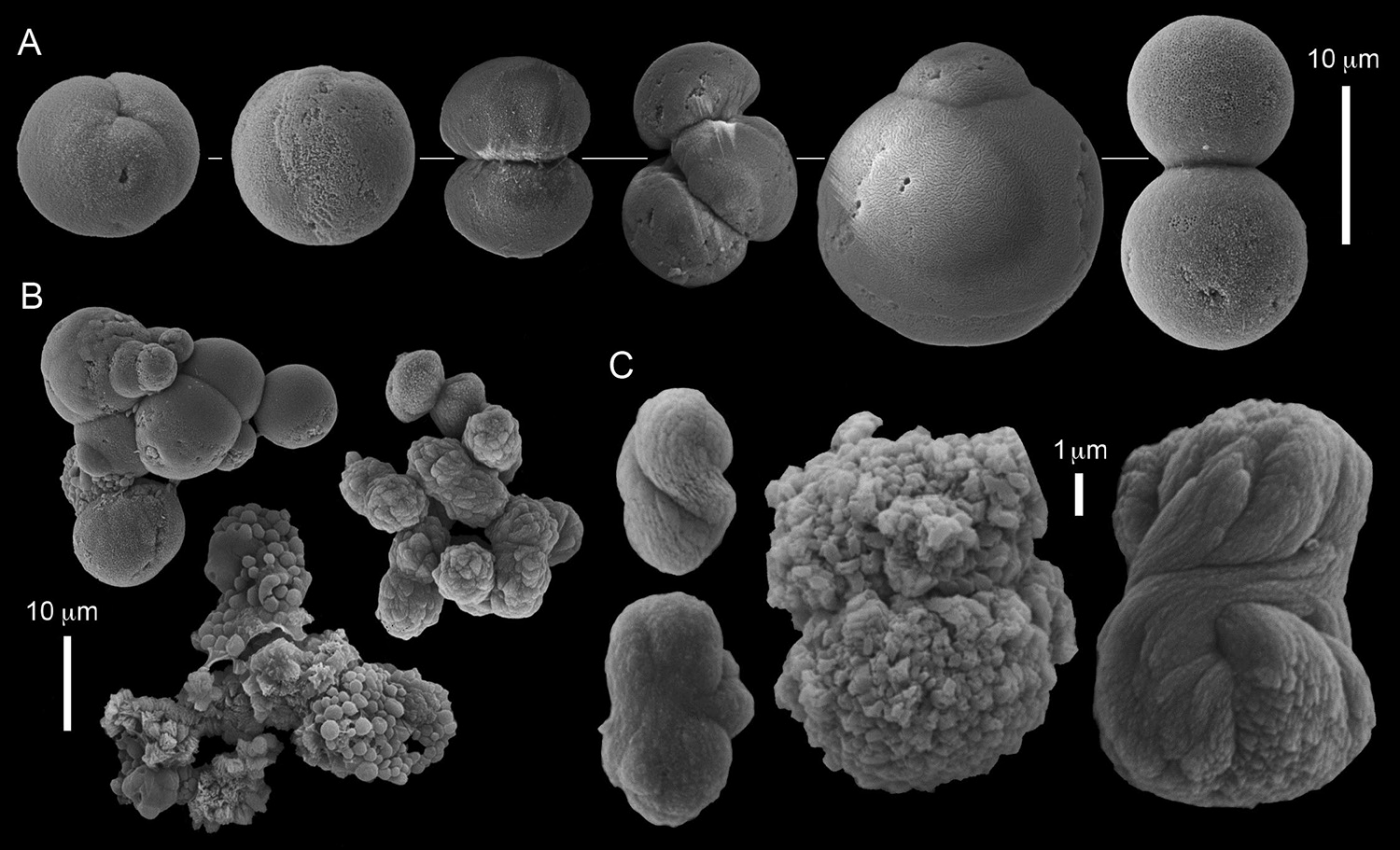
*Conglomeratusclera coerulea* (May, 1898), ZMTAU Co 35707. **A** Spheres and double spheres. **B** conglomerate of spheres and spherules **C** twisted dumbbells.

**Figure 12.**
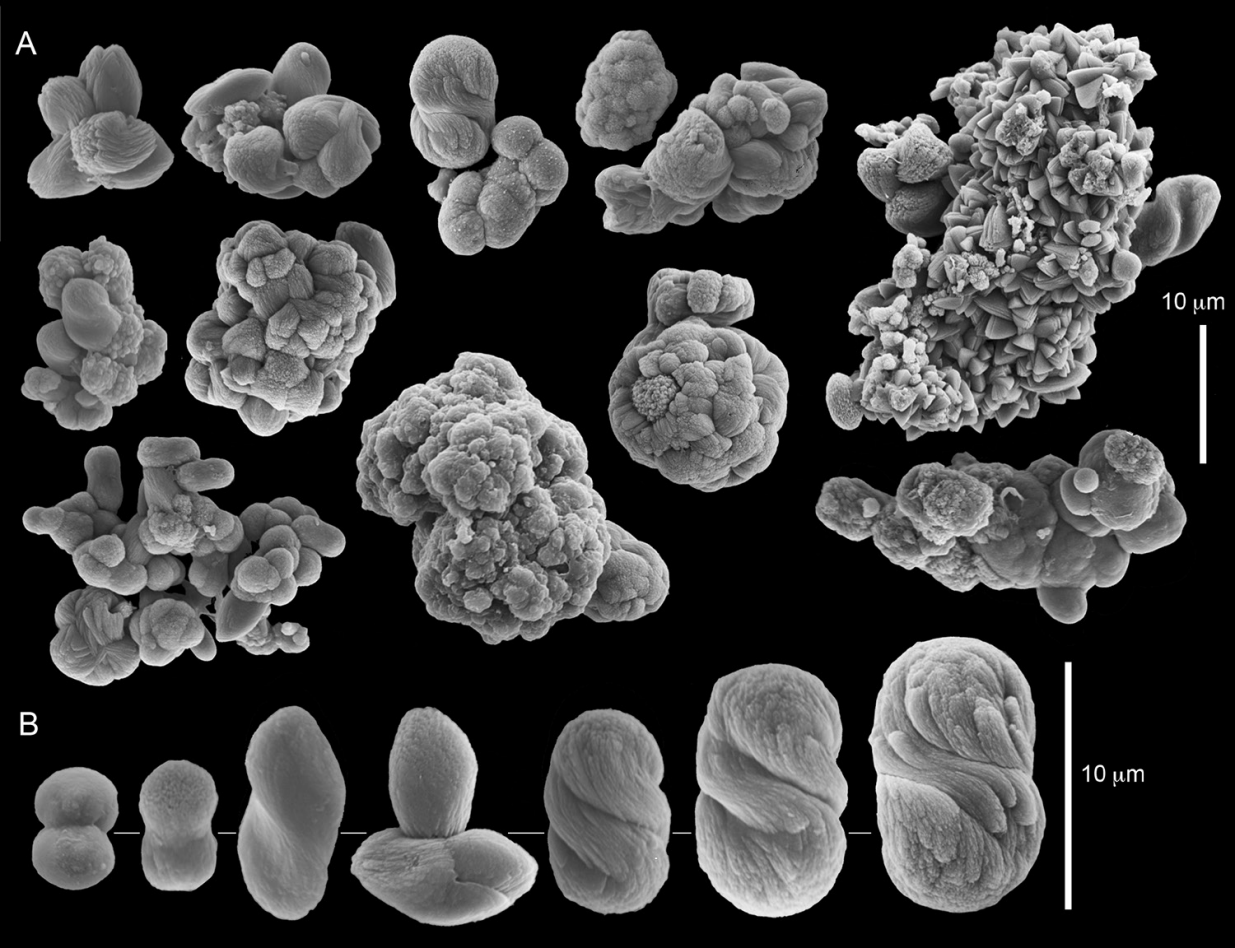
*Conglomeratusclera coerulea* (May, 1898), ZMTAU Co 35710. **A** conglomerate sclerites composed of spherules and twisted dumbbells **B** double heads and twisted dumbbells.

**Figure 13.**
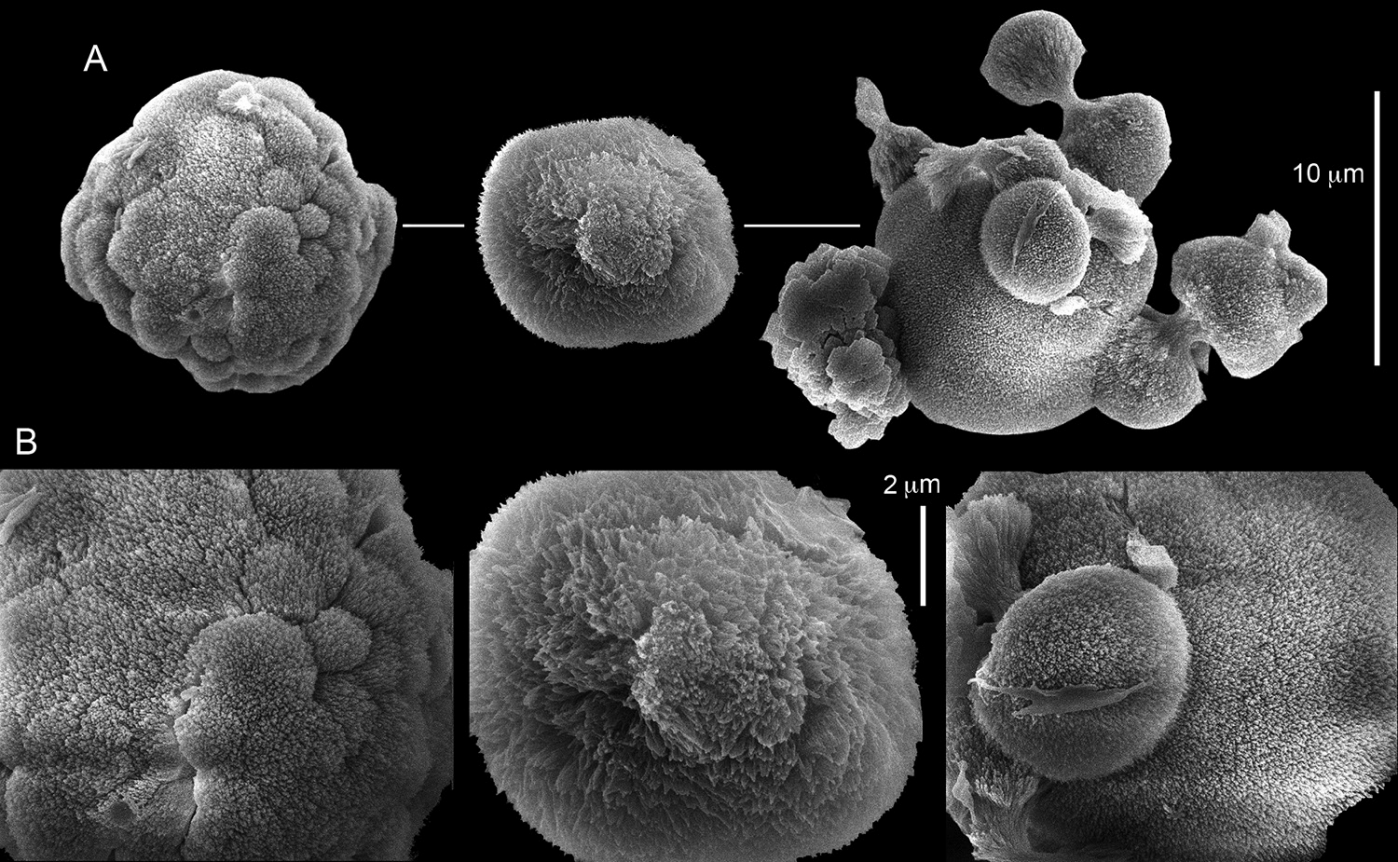
*Conglomeratusclera coerulea* (May, 1898), ZMTAU Co 35712. **A–B** bristly surface of spherules and spheres.

**Figure 14.**
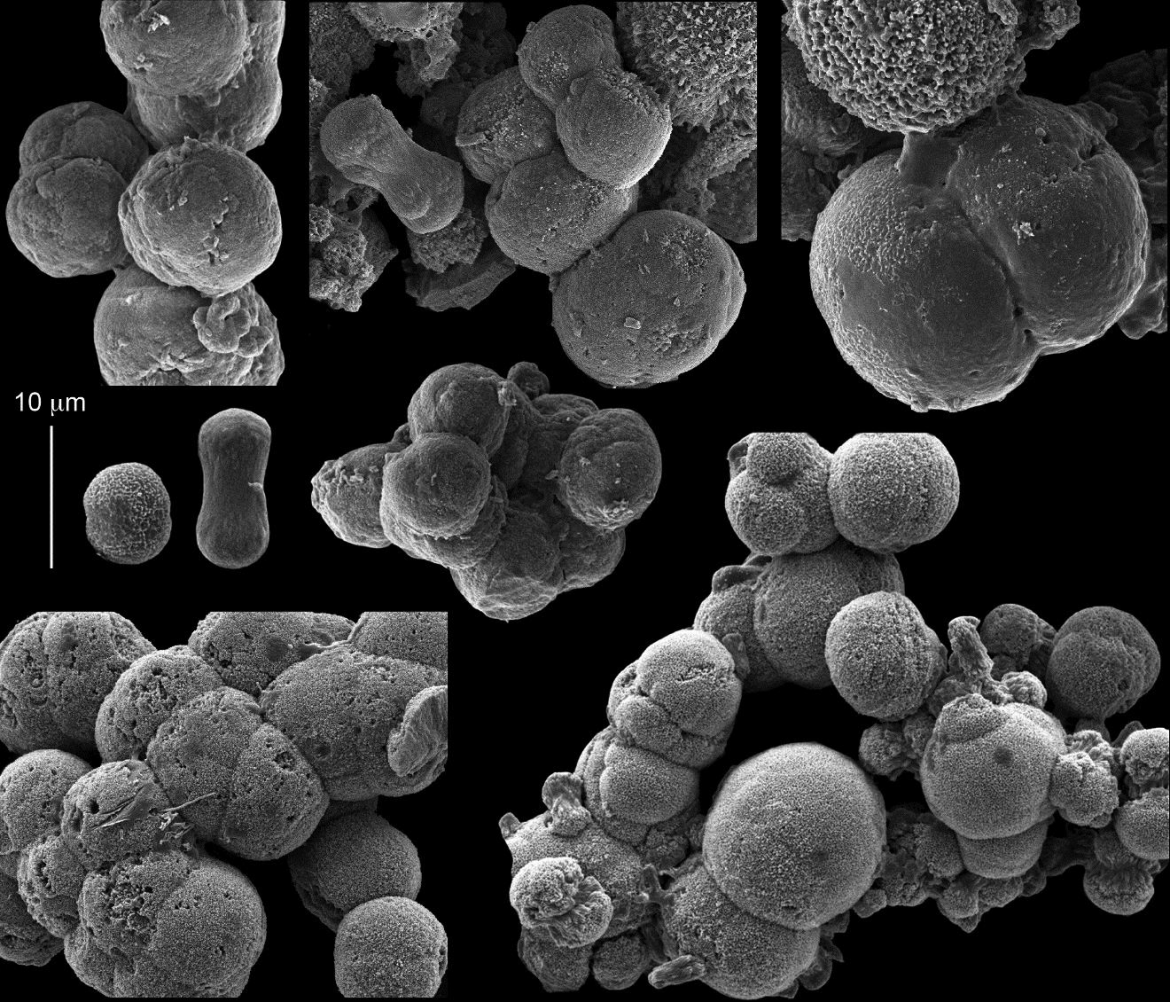
*Conglomeratusclera coerulea* (May, 1898), ZMTAU Co 35131. Conglomerate sclerites composed of spheres and spherules, individual elongate double head.

**Figure 15.**
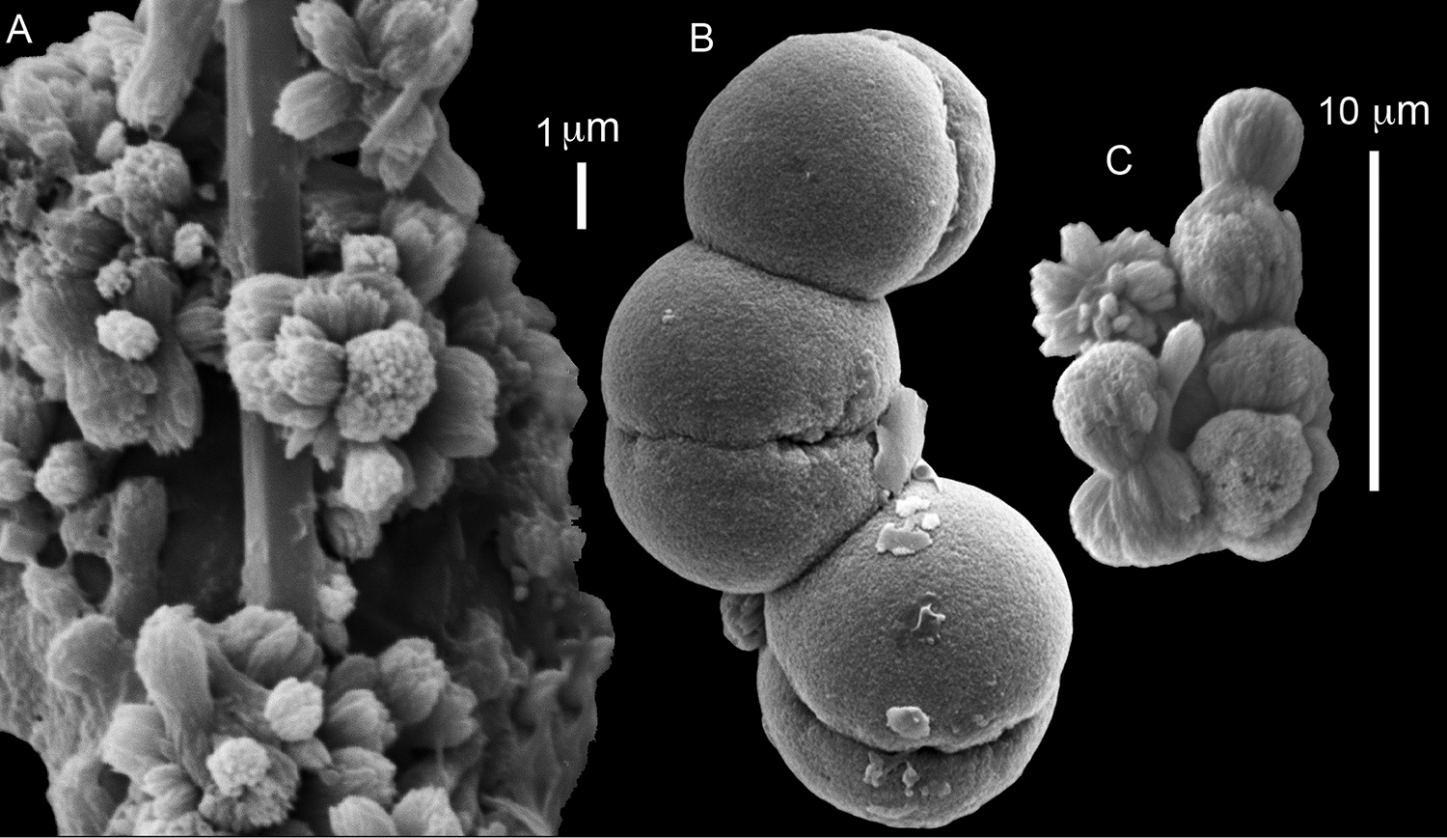
*Conglomeratusclera coerulea* (May, 1898), ZMTAU Co 36129. **A** conglomerate of bristly dumbbells **B** conglomerate of spheres **C** conglomerate of dumbbells. Scale at **A** also applies to **B**.

**Figure 16.**
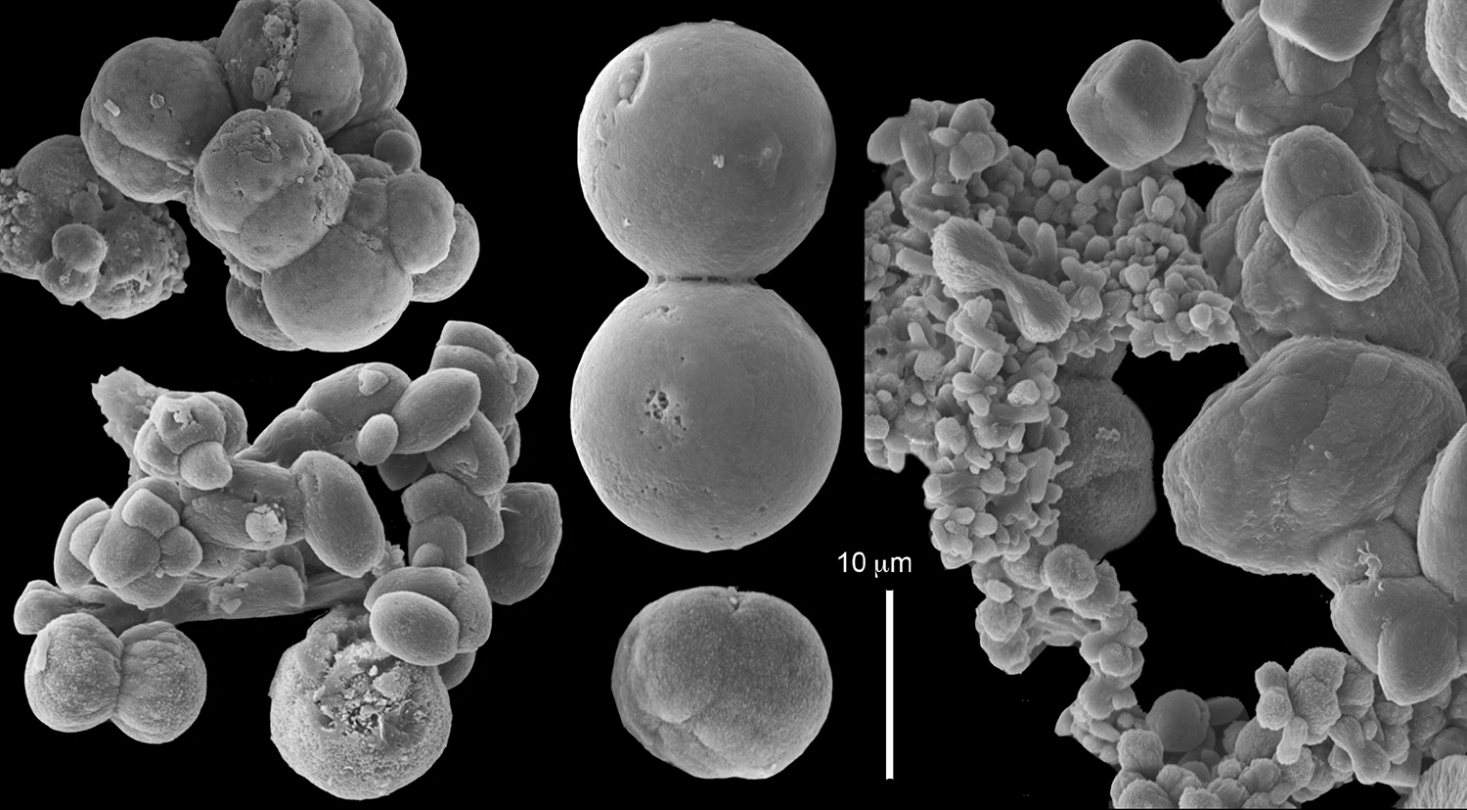
*Conglomeratusclera coerulea* (May, 1898), ZMTAU Co 36013. Conglomerate sclerites composed of spheres, spherules and dumbbells, individual sphere.

**Figure 17.**
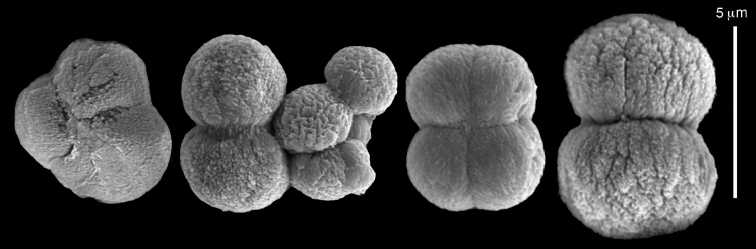
*Conglomeratusclera coerulea* (May, 1898), BMNH 1933.3.13.175. Spheres and double heads with bristly surfaces. Some double heads joined to form a more cross-like sclerite.

The sclerites of the colonies noted above featured the full array of morphologies, mostly corresponding to that of the syntypes (Figures 2–3). To demonstrate the vast variation in shape and size of the sclerites, SEM images of sclerites of several sequenced colonies are presented for the Taiwan material: ZMTAU Co 35692 (Figure 7), Co 35737 (Figure 8), Co 35765 (Figure 9), Co 35709 (Figure 10), Co 35707 (Figure 11), Co 35710 (Figure 12), and Co 35712 (Figure 13), Yonaguni: ZMTAU Co 35131 (Figure 14) and Madagascar Co 36129 (Figure 15), and Co 36013 (Figure 16). Figures 7–16 demonstrate the morphological variability of the sclerites, with all being conglomerates comprised mainly of spheres and spherules and occasionally dumbbells. The SEM images revealed that their outer surface is sometimes bristly (Figures 7B–D, 8B, 13A–B, 14, 15A, 17) but commonly rather smooth (Figures 8A, 10A–B, 11A–B, 15B, 16). It is interesting to note that the spheres are sometimes embedded in a calcareous lamella-like structure (Figure 7A). Dumbbells were revealed in some colonies (Figures 8B, 12B, 14, 15C, 17) as well as twisted dumbbells (Figures 9, 11C, 12B). Similarly, as noted above for the syntypes, the above SEM images indicate that the aggregates tend to disintegrate during sclerite preparation and therefore their actual dimensions cannot be determined.

The molecular results indicate that despite the differences in pinnule count and sclerite morphology, all the colonies should be assigned to the same species (Figure 6). Consequently, the pinnule count is of no diagnostic value for species delineation within *Conglomeratusclera*. *C. coerulea* thus accommodates colonies with one row of pinnules on the margins of the polyp tentacles, but featuring a remarkable range of pinnule numbers (see above). In addition, the variable sclerite morphologies found in the different colonies (Figures 7–16) both encompass and exceed the range observed among the syntypes of *C. coerulea* (Figures 2A–B). The current results provide further support for the recent findings of McFadden et al. (2017) who argue that the pinnule count used in the taxonomy of Xeniidae, explicitly in the genus *Ovabunda* (see references in Halàsz et al. 2014), is not indicative of species boundaries. It should be noted that in contrast to the relatively uniform morphology of *Ovabunda* sclerites recorded across the four genetic clades presented by McFadden et al. (2017), colonies of *C. coerulea* exhibit an unprecedented and bewildering array of sclerite morphologies (Figures 2A, B, 7–16).

Material that was examined, but not sequenced, comprised both freshly collected colonies and museum specimens. Their colony and polyp morphologies, including the pinnule counts, are in agreement with the findings presented above. Noteworthy are some colonies for which SEM or light microscopy could not detect any sclerites. There are several suggested reasons for this: (1) actual lack of sclerites; (2) their low incidence which led to a failure to detect them by SEM; or (3) preservation procedures, such as acidic conditions that may have caused sclerite dissolution.

**Figure 18.**
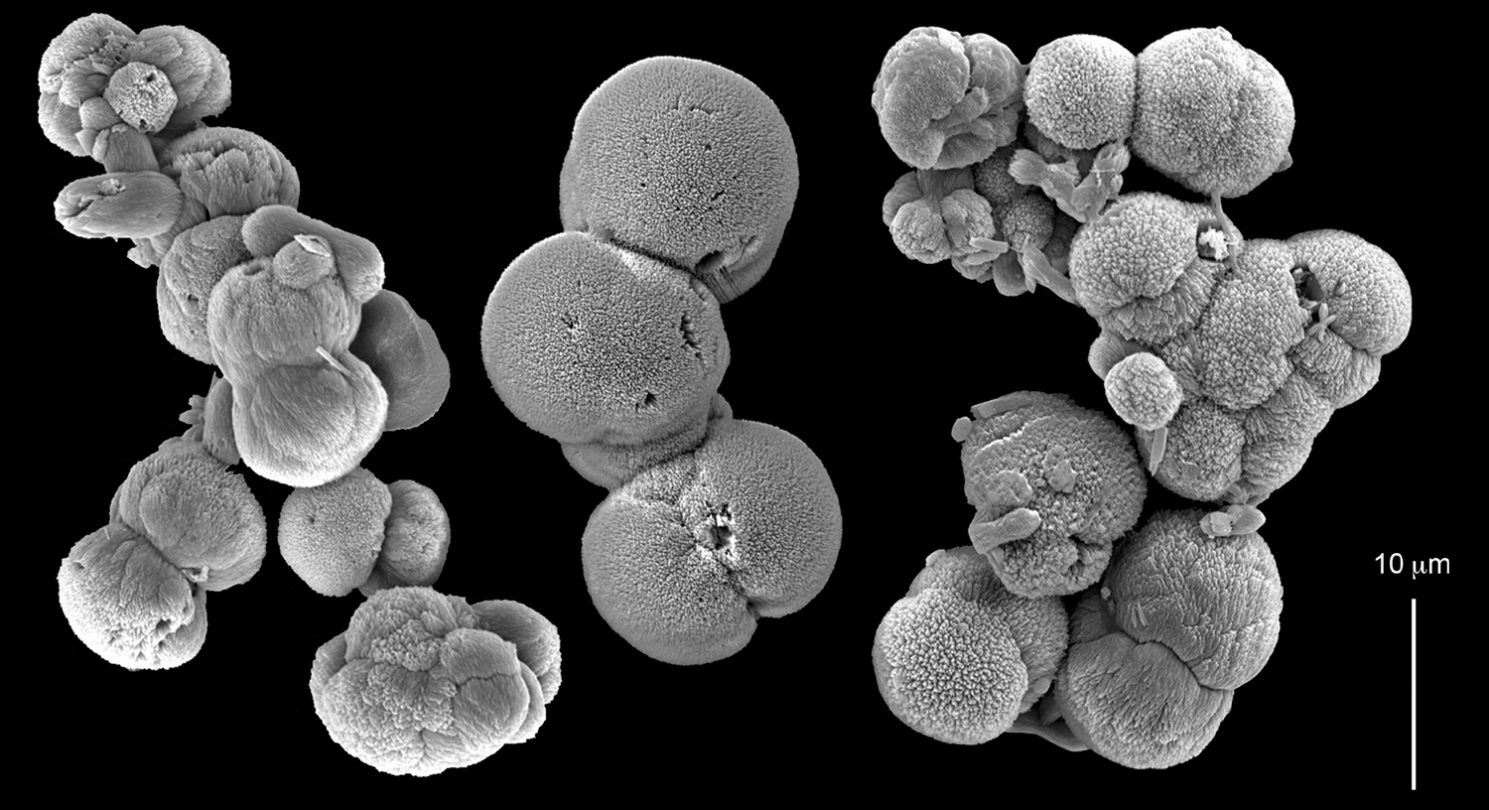
*Conglomeratusclera coerulea* (May, 1898), BMNH 1912.2.24.65. Conglomerate sclerites composed of spheres and spherules. Bristly surface is noted.

**Figure 19.**
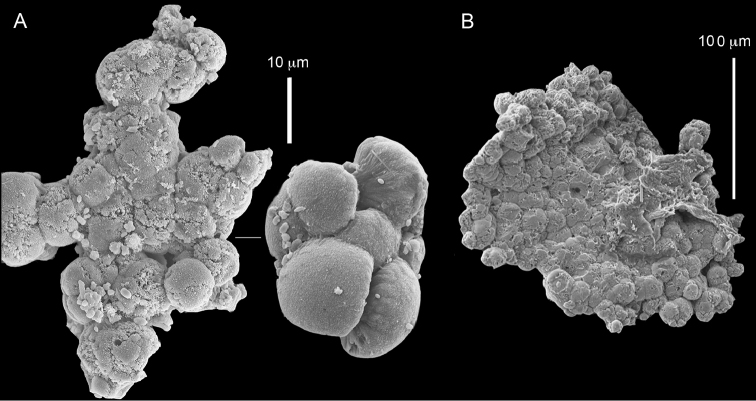
*Conglomeratusclera coerulea* (May, 1898), BMNH 1912.2.24.66. **A** conglomerate sclerites composed of spheres and spherules **B** plate-like conglomerate of spherules.

**Figure 20.**
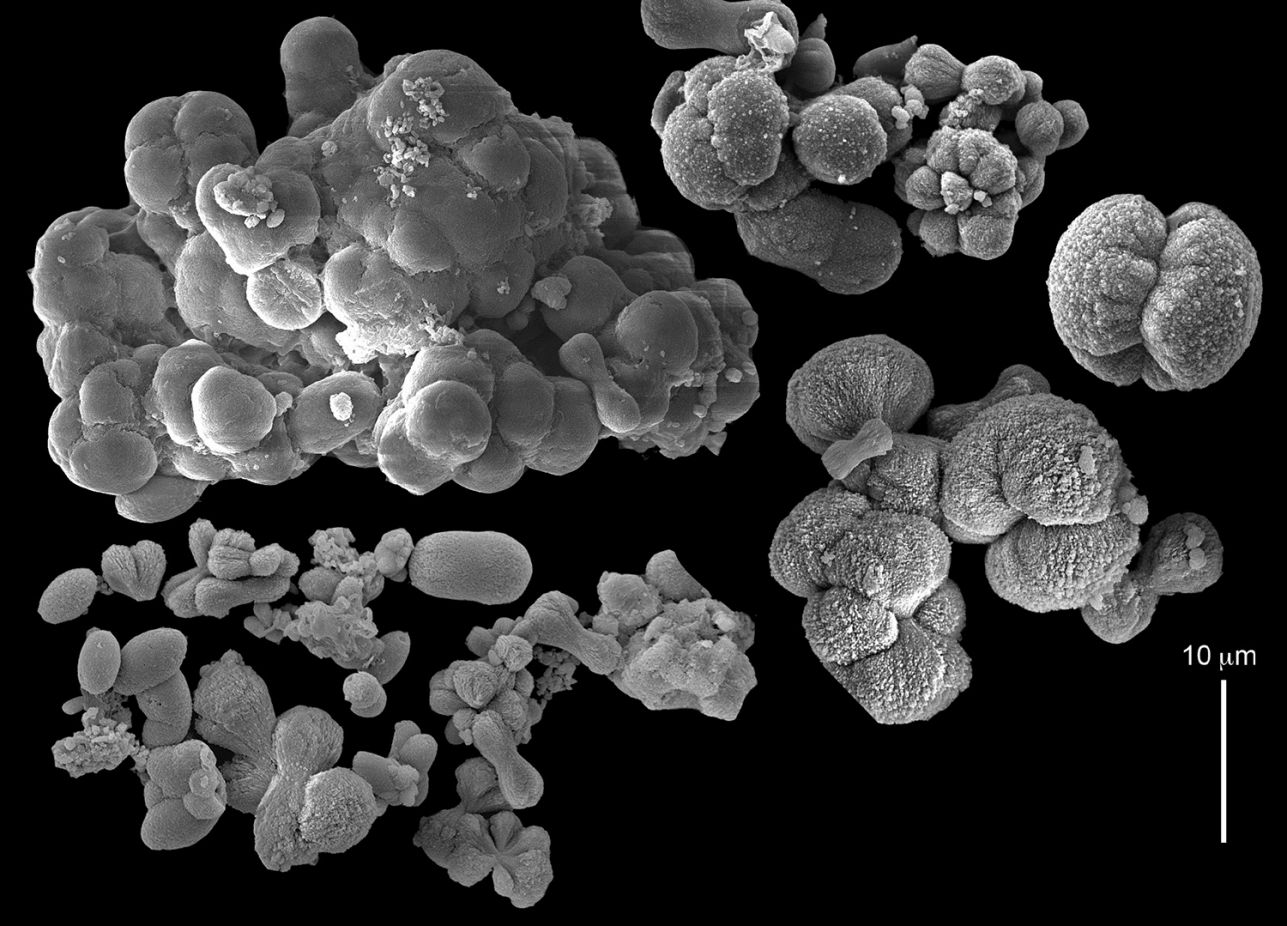
*Conglomeratusclera coerulea* (May, 1898), BMNH 1912.2.24.67. Conglomerate sclerites composed of spheres, spherules and dumbbells. Bristly surfaces of some sclerites is noted.

**Figure 21.**
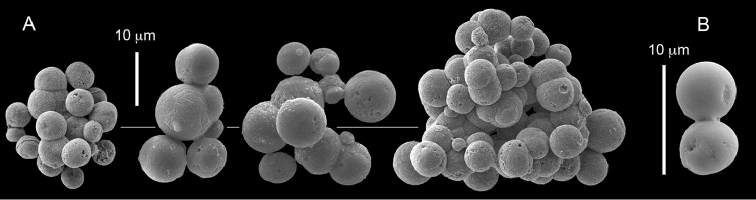
*Conglomeratusclera coerulea* (May, 1898), BMNH USNM 60795. Conglomerate sclerites of spheres and spherules.

**Figure 22.**
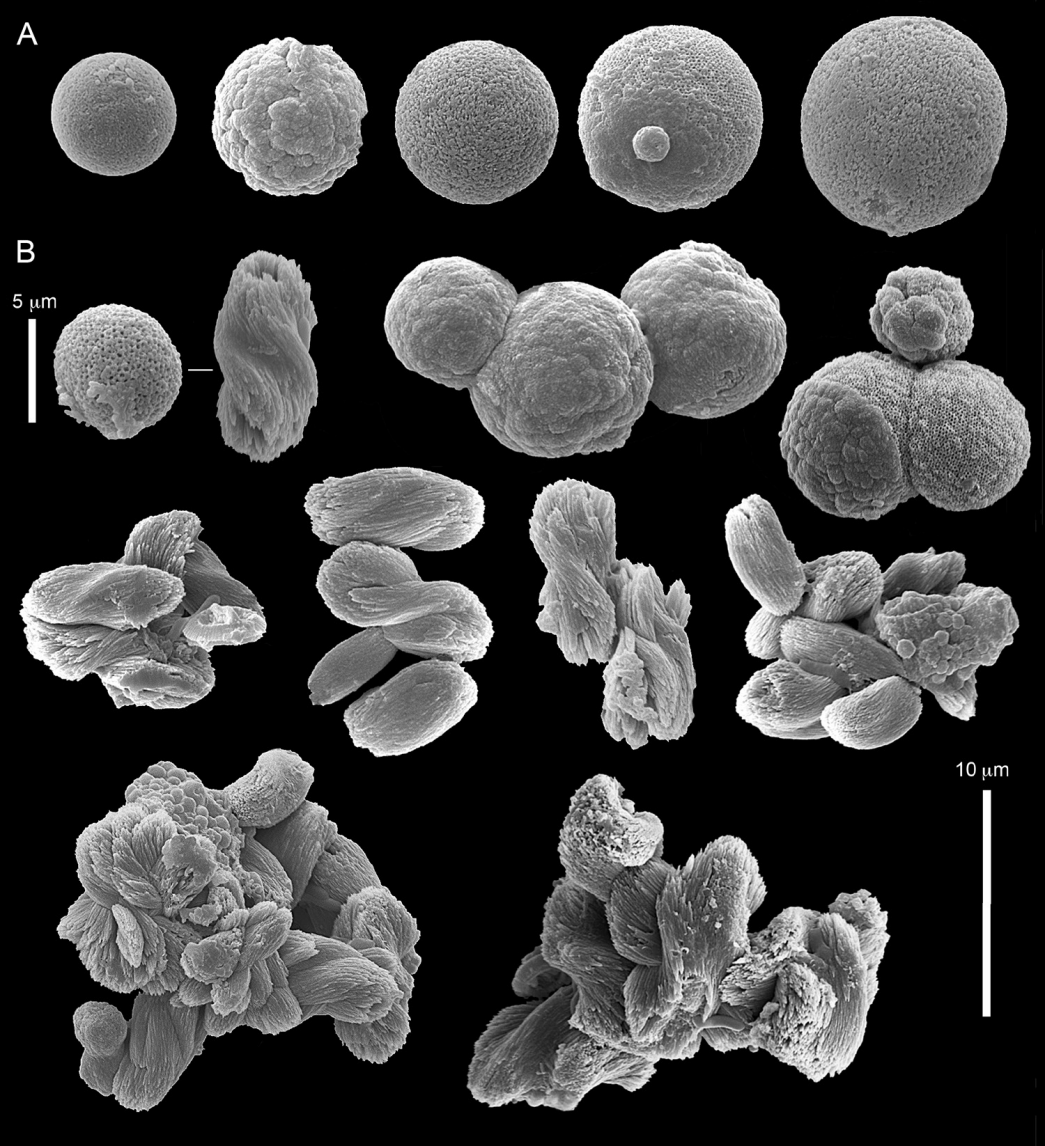
*Conglomeratusclera coerulea* (May, 1898), RMNH Coel 42160. **A** spheres **B** sphere, twisted dumbbell and conglomerate sclerites composed of spheres and twisted dumbbells.

**Figure 23.**
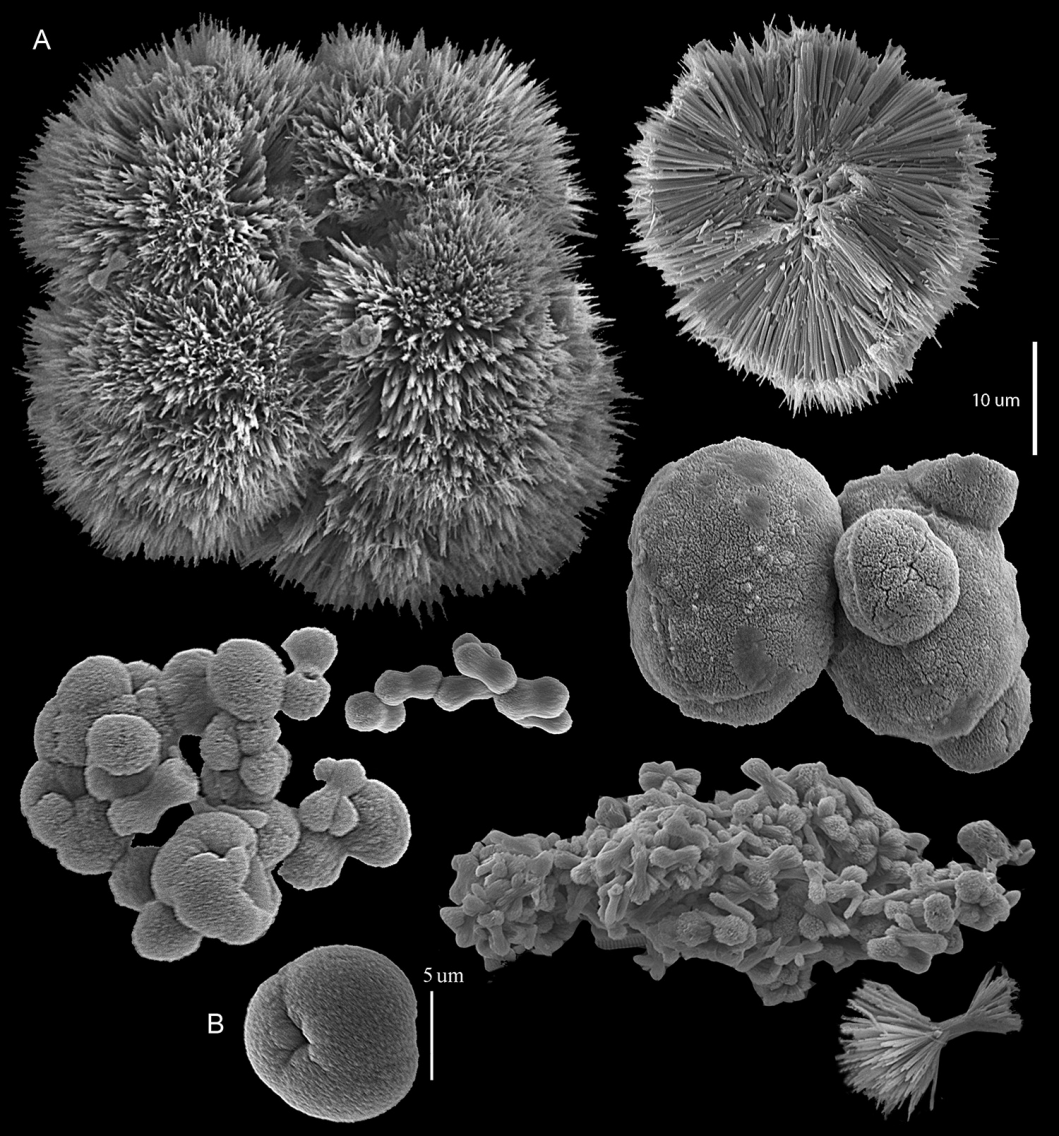
*Conglomeratusclera coerulea* (May, 1898), RMNH Coel 42161. Conglomerate sclerites composed of spheres and spherules. Bristly surface of spheres is noted.

**Figure 24.**
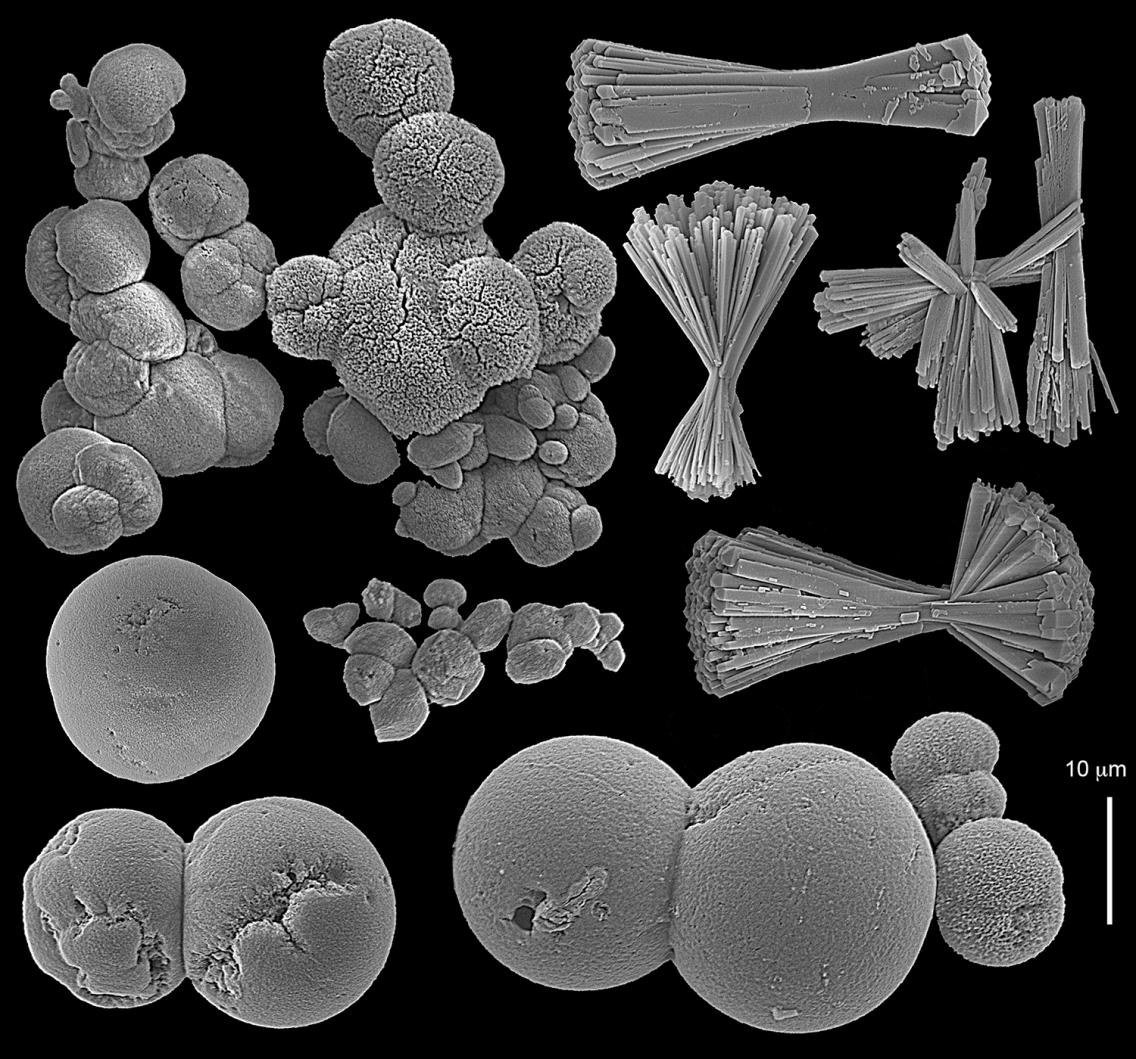
*Conglomeratusclera coerulea* (May, 1898), RMNH Coel 42161. Conglomerate sclerites composed of spheres and spherules. Some crystalline bundles are presented.

**Figure 25.**
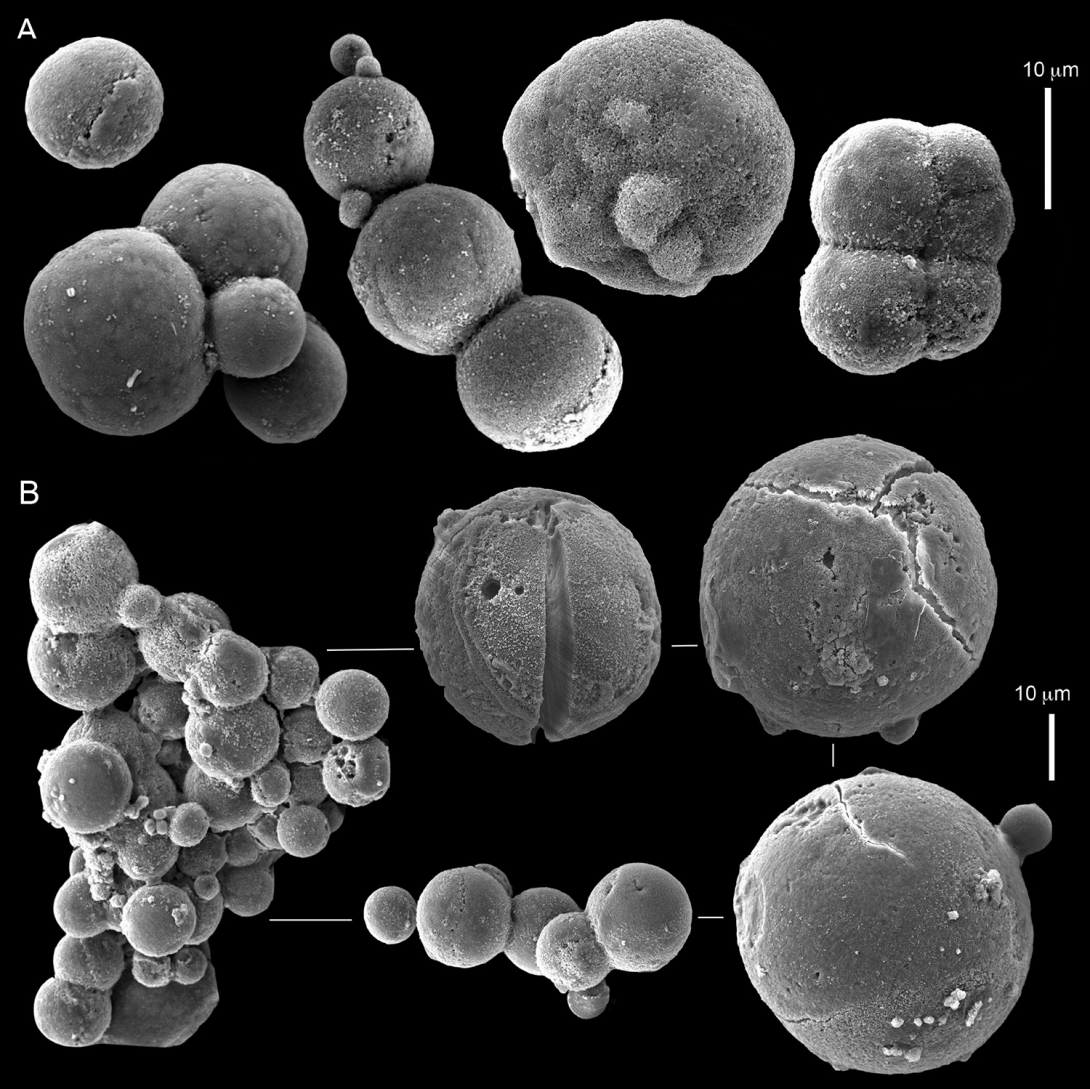
*Conglomeratusclera coerulea* (May, 1898), RMNH Coel 42162. **A–B** Conglomerate sclerites composed of spheres and spherules, some double heads joined to form a more cross-like sclerite.

The museum material examined included colonies from the BMNH, all collected from the western Indian Ocean (see above). Some of the colonies were originally identified by L.M.I. Macfadyen as *Cespitularia coerulea* (BMNH 1912.2.24.66 and 1933.3.13.175; Figure 17), *C. mollis* (BMNH 1933.313.177), *C. taeniata* (BMNH 1912.2.24.65, 1933.5.3.301 and 1933.3.13.176) and *Cespitularia wisharti* Hickson, 1931 (BMNH 1934.3.28.10). These BMNH colonies feature one row of 8–13 pinnules along each side of their tentacles and the morphology of their sclerites corresponds to that of *Conglomeratuscslera coerulea* [e.g., BMNH 1912.2.24.65 (Figure 18), 1912.2.24.66 (Figure 19), 1912.2.24.67 (Figure 20)]. The morphological examination therefore indicates that the BMNH material should be assigned to the above species. The sclerites of the colony from the Great Barrier Reef, Australia, USNM 60795 (Figure 21), as well as those of USNM 54000 and 54003 (sclerites not shown), collected in Madagascar, similarly confirmed them to be *C. coerulea*. The RMNH material too revealed colonies that have now been assigned by us to *C. coerulea*, featuring one row of 8–16 pinnules along each side of their tentacles as well as sclerites: RMNH Coel 42160 (Figure 22), Coel 42161 (Figures 23–24) and RMNH Coel 42162 (Figure 25). These images reveal spheres, either in a conglomerated form or individuals (Figures 22–25), and in other colonies mostly twisted dumbbells, either aggregated or individual (Figure 22). Interestingly, some crystalline bundles were noted among the spheres (Figure 24).

######## Distribution.

Kenya; Zanzibar; Tanzania; Glorioso Islands; Mauritius; Seychelles; Mayotte; Taiwan; Philippines; Japan (Tanabe, Wakayama, Shikoku); Ryukyu Archipelago; Indonesia.

####### Conglomeratusclera robusta

Taxon classificationAnimaliaAlcyonaceaXeniidae

(Tixier-Durivault, 1966)

**Figure 26.**
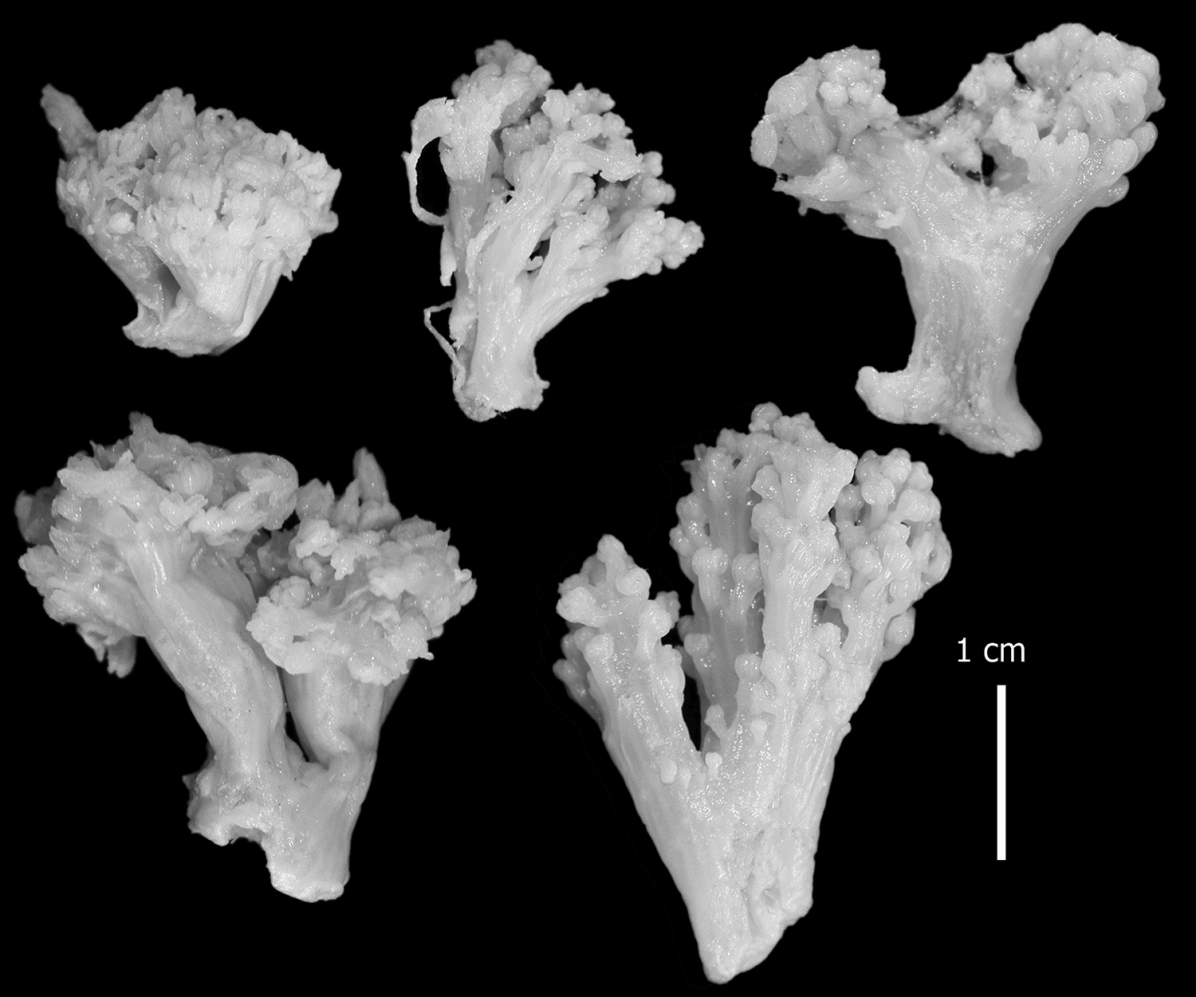
*Conglomeratusclera robusta* (Tixier-Durivault, 1966), syntypes MNH00000167.

**Figure 27.**
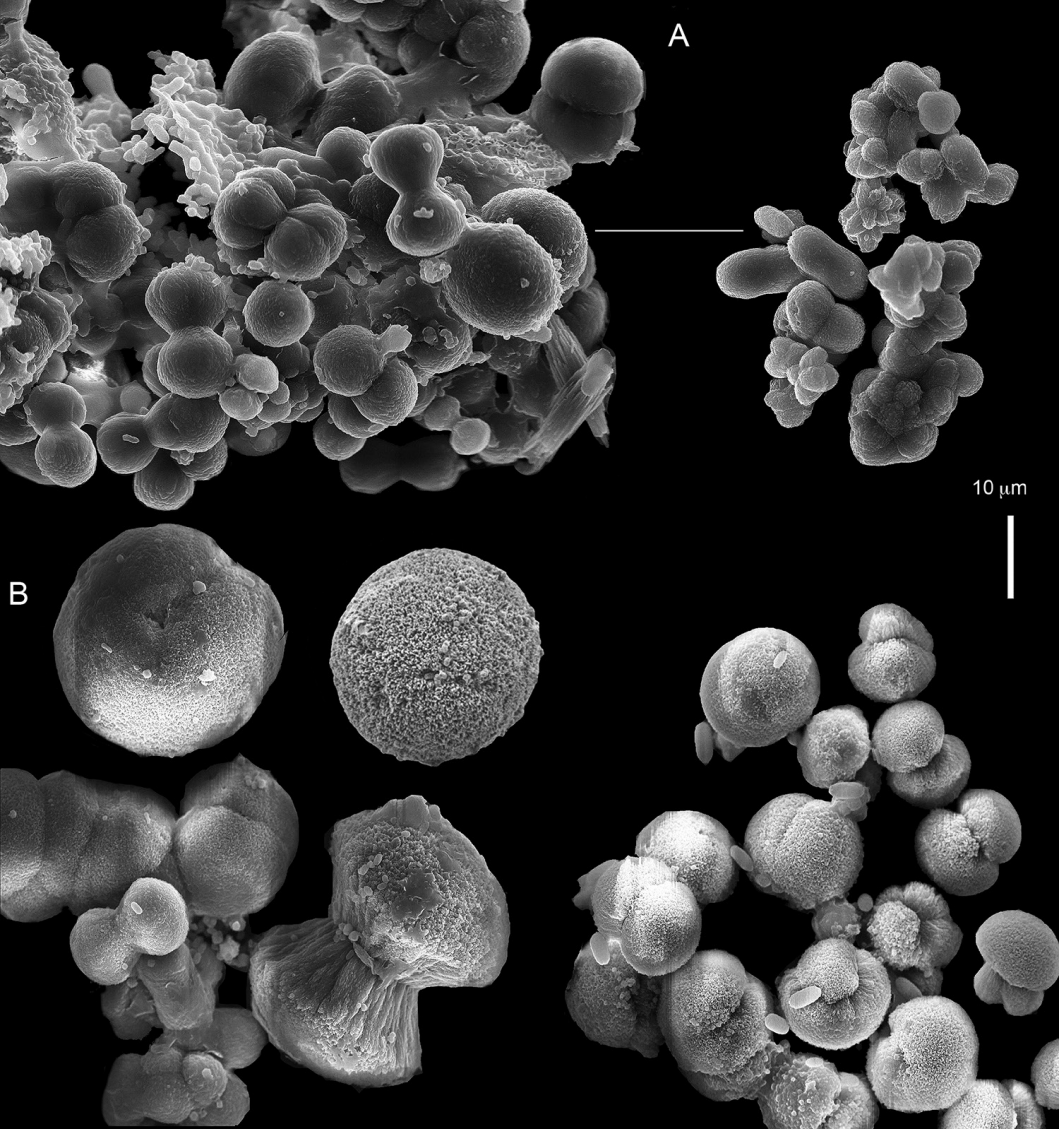
*Conglomeratusclera robusta* (Tixier-Durivault, 1966). **A** Conglomerate sclerites composed of spheres and spherules **B** Spheres, dumbbell, and conglomerate sclerites.

**Figure 28.**
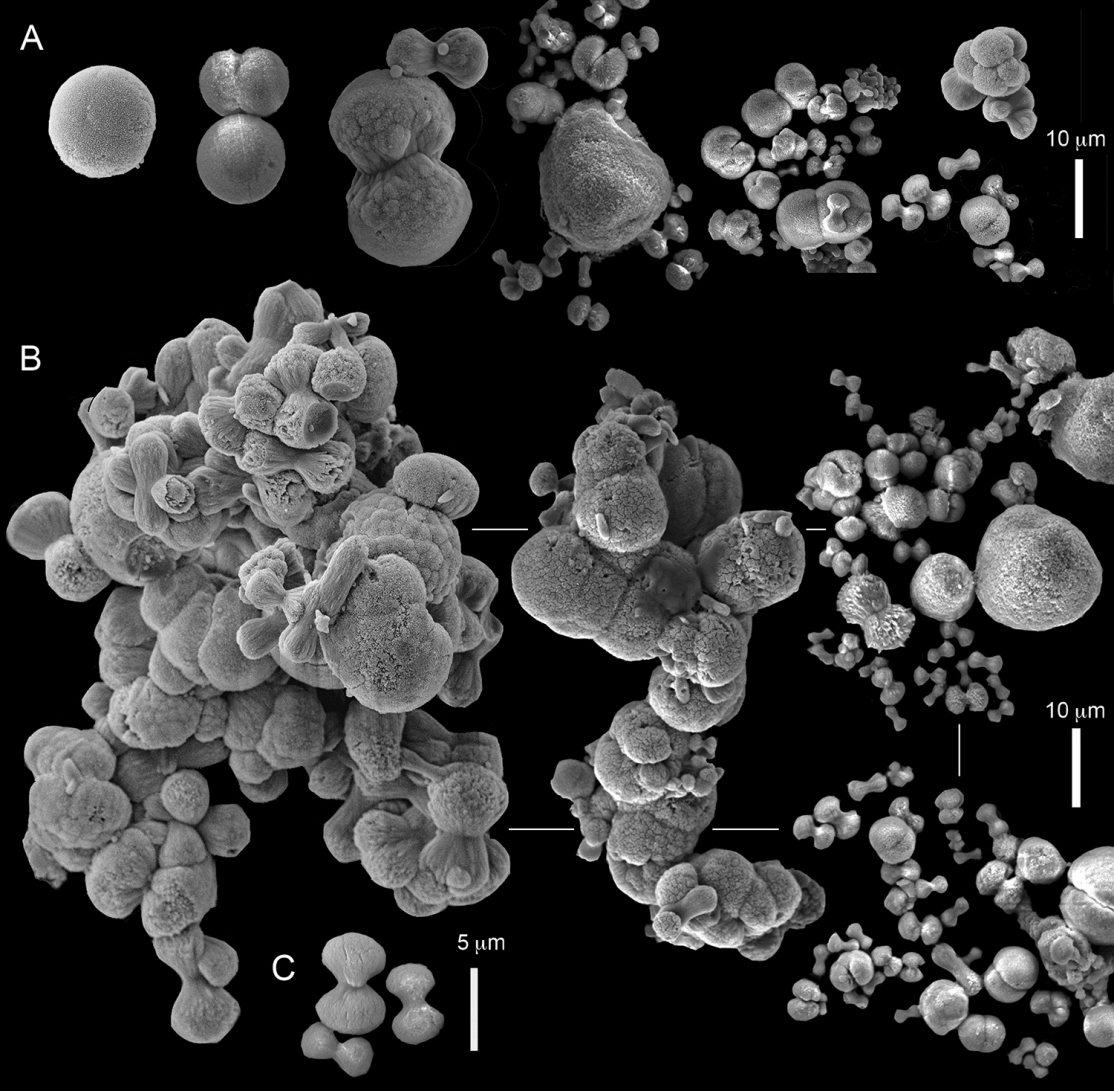
*Conglomeratusclera robusta* (Tixier-Durivault, 1966) RMNH Coel 38672. **A–B** conglomerate sclerites composed of spheres, spherules, and dumbbells. **C** dumbbell sclerites.

Figures 26 , 27 , 28

Cespitularia robusta Tixier-Durivault, 1966: 335–356; Janes 2008: 604–605.

######## Description.

Examination of the type material of *Cespitularia robusta* Tixier-Durivault, 1966 (MNH00000167) revealed five colonies (Figure 26), all in agreement with their original description. The tentacles bear two rows of pinnules along each side with an indication of a third row; the outermost row features 12–15 pinnules. The sclerites depicted in the original description are spheres and spherules, also in the form of aggregates (p. 356: fig. 321 C–N). The SEM images of the sclerites (Figure 27) reveal morphologies similar to those found in *C. coerulea* (see above), and therefore led us to assign the species to *Conglomeratusclera* n. gen instead of *Cespitularia*. Subsequent examination of *C. robusta* (RMNH Coel 38672), identified by Janes (2008), similarly confirmed his findings but based on the sclerite SEM images of that colony (Figure 28), the generic assignment is likewise changed to *Conglomeratusclera*.

The colonies assigned by us to *C. coerulea* feature one row of pinnules along the margins of the tentacles, whereas *C. robusta* has two rows. In order to determine whether a difference in pinnule-row count is indeed diagnostic for species delineation in *Conglomeratusclera*, corresponding fresh colonies with two pinnule-rows should be sequenced. Therefore, for the time being only the generic status of *C. robusta* is changed, making it the second species in the new genus.

######## Distribution.

Mayotte; Aride Island, Seychelles.

####### Caementabunda gen. n.

Taxon classificationAnimaliaAlcyonaceaXeniidae

http://zoobank.org/899AF711-D0A6-43F7-A3A6-75F481DF29A6

######## Type species.

*Cespitularia simplex* Thomson & Dean, 1931

######## Diagnosis.

Colonies quite flaccid with a distinct but short encrusting base bearing primary lobes, sometimes divided into secondary ones. Non-retractile monomorphic polyps found on the lobes and occasionally down on some parts of the base. The spherical-oval sclerites are composed of a myriad of densely packed chip-like microscleres. Zooxanthellate.

######## Etymology.

The generic name refers to the microstructure of the sclerites, which are composed of multitudes of microscleres, resembling aggregates of cement chips. The name is derived from the Latin *caementum*, cement, and *abunda* meaning copious. Gender feminine.

####### Caementabunda simplex

Taxon classificationAnimaliaAlcyonaceaXeniidae

(Thomson & Dean, 1931)

Figures 5C–D , 29 , 30 , 31 , 32 , 33 , 34 , 35 , 36 , 37

Cespitularia simplex Thomson & Dean, 1931: 33–34; Macfadyen 1936:27; Verseveldt 1971: 62; Janes 2008: 606–608; Janes 2013: 198 (listed only); McFadden et al. 2014: 249 (listed only), Cespitularia turgida Verseveldt, 1971: 61–62.

######## Material.

**Syntype: INDONESIA**: ZMA 2344, Siboga Exped., Sta. 40, 12 m depth, Kawassang**. Other material**: **SEYCHELLES**: RMNH Coel 38673, Southern coast of Aride I. (04°13'S; 55°40'E), <20 m depth, 18 December 1992; **MADAGASCAR**: RMNH Coel 6697, Nosy Be, west of Andilina, 24 August, 1967, 20 m depth; RMNH Coel 42168, Stn. 22, 21 December 1999; RMNH Coel 42169; **PHILIPPINES**: Cebu Strait Exped., Sta. CEB. 1, Cebu Strait, Olango Channel, east side of Olango Is., USNM 60493, Sulu Archipelago, 6°07'N, 121°00'E, R/V Albatross; **AUSTRALIA**: USNM 60794, Flinders Reef, Great Barrier Reef, November 1981; BMNH 1934.3.28.8, Great Barrier Reef Exped., Sta. 10, dredge, 22 February 1929; 1982.11.17, Great Barrier Reef, Flinders Reef, South Coral Sea, southern outer slope, 10–15 m depth, coll. Z. Dinesen; BMNH 1982.11.18, similar details; **JAPAN**: ZMTAU Co 31642, off Danno, Yonaguni Is., Ryukyu Archipelago, 24°27'N, 122°57'E, 15 m depth, coll. Y. Benayahu, 13 November 1992; ZMTAU Co 31638, Mao Cave, Shimoji Is., Ryukyu Archipelago, 10 m depth, coll. Y. Benayahu, 19 November 1992; ZMTAU Co 35120, Umabanazaki Point, Yonaguni Is., Ryukyu Archipelago, 8–12 m depth, coll. Y. Benayahu, 3 June 2010; **MADAGASCAR**: ZMTAU Co 36057, three specimens; ZMTAU Co 36076, 4 Frères, 13°00.142'S, 48°29.099'E, 6–14 m depth, coll. Y. Benayahu, 2 December 2012; ZMTAU Co 36065, 4 Frères, 12°59.655'S, 48°29.248'E, 4–15 m depth, coll. Y. Benayahu, 1 December 2012, four specimens; ZMTAU Co 36115, Ronald Point, Nosy Be, 13°23.530'S, 48°00.143'E, 19–27 m depth, coll. Y. Benayahu, 3 December 2012; ZMTAU Co 36122, Ronald Point, Nosy Be, 13°29.032'S, 47°58.721'E, 2–4 m depth, coll. Y. Benayahu, 03 December 2012, two specimens; ZMTAU Co 36127, details as before; **TAIWAN**: Co 33021, Chaikou, Green Is., Taiwan, 22°40'40"N, 121°28'20"E, 3–6 m depth, coll. Y. Benayahu, 13 July 2005; ZMTAU Co 35715, Shihlang, Green Is., 22°39.425'N, 121°28.399'E, 8–12 m depth, coll. Y. Benayahu, 3 September 2012; ZMTAU Co 33022, Lomenyen, Green Is., 22°40'56"N, 121°30'06"E, 3–25 m depth, coll. Y. Benayahu, 12 July 2005; ZMTAU Co 35713, details as before, three specimens; ZMTAU Co 35701, details as before, four specimens; ZMTAU Co 35757, Shihlang, Green Is., 22°39.425'N, 121°28.399'E, 7–10 m depth, coll. Y. Benayahu, 5 September 2012, four specimens.

######## Description.

**Figure 29.**
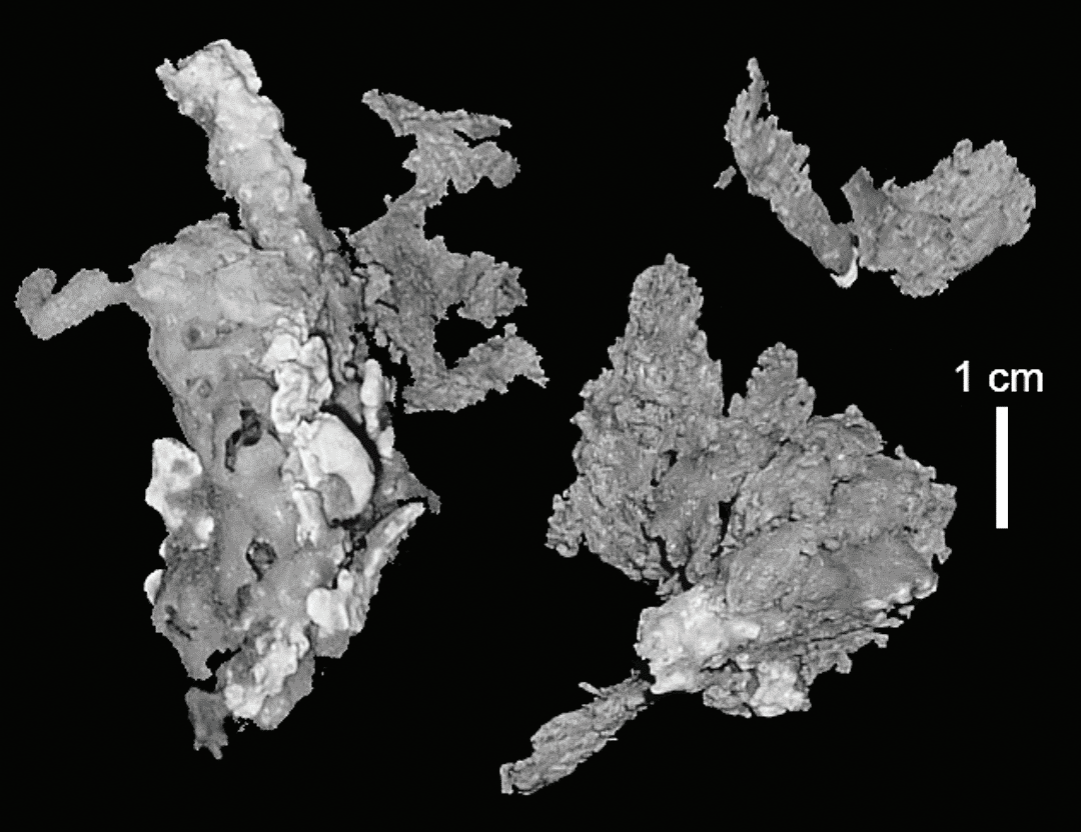
*Caementabunda simplex* (Thomson & Dean, 1931) Syntypes, ZMA 2344.

**Figure 30.**
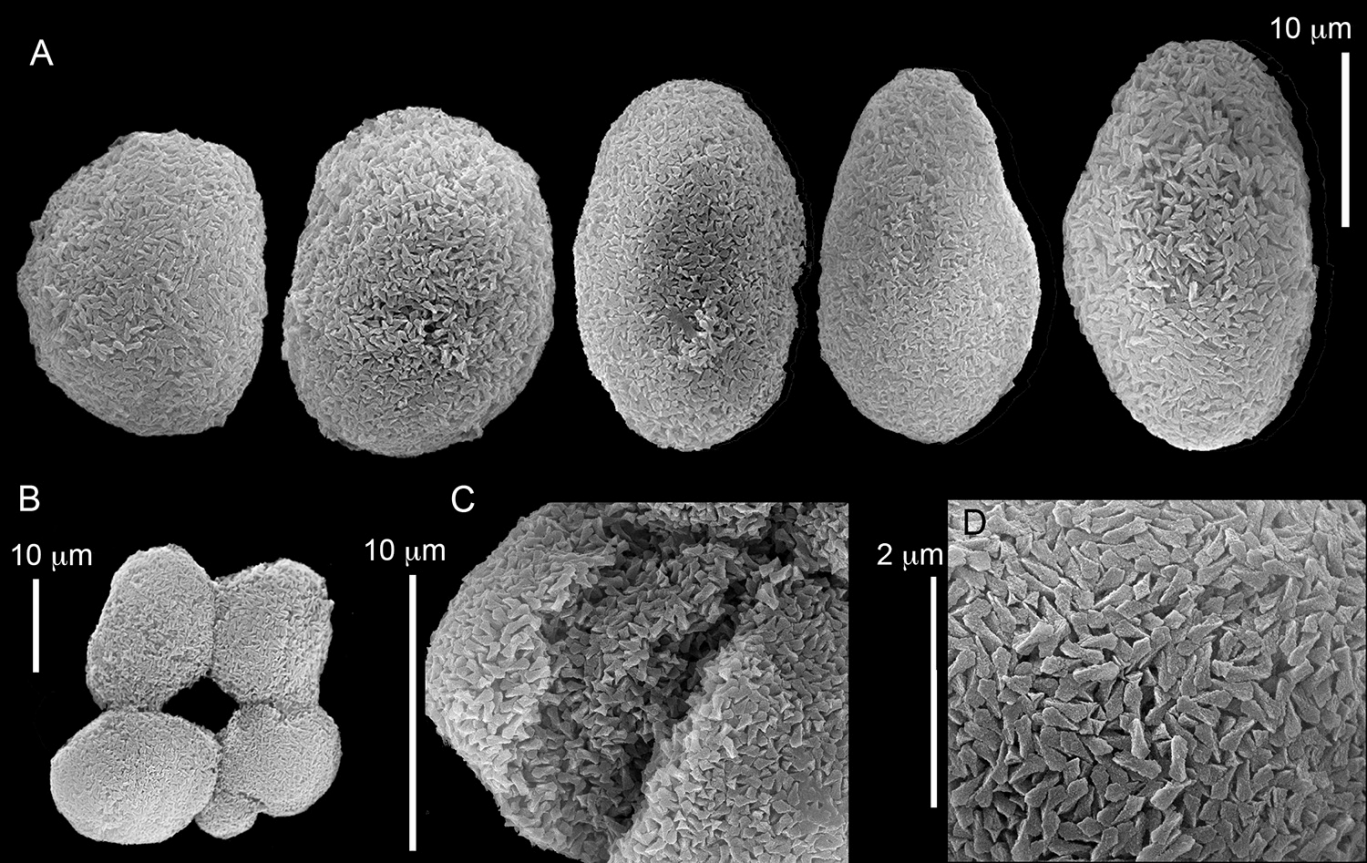
*Caementabunda simplex* (Thomson & Dean, 1931) Syntypes, ZMA 2344**. A** spheroid sclerites **B** cluster of spheroid sclerites **C** fractured spheroid showing densely packed chips-like microscleres **D** densely packed chips-like microscleres of spheroid’s surface.

The syntype RMNH Coel 2344 consists of three encrusting lobed colonies attached to calcareous fragments. The largest syntype is 3 cm high by 5 cm wide, the second 1.5 by 2.5 cm, and the third 2 by 3.5 cm (Figure 29). The finger-like lobes feature non-retractile polyps, some of which are found on the colony base. The polyp body is up to 2.8 mm long and the tentacles are up to 1.0 mm long. The tentacles bear one row of 12–14 pinnules along each of their margins. The short pinnules are closely set, with no space between adjacent ones. The preserved colonies are brown-beige. Sclerites are highly abundant and found in all parts of the colony. Under the light microscope they are ovoid or pear-shaped as fully confirmed by SEM (Figure 30A), measuring up to 0.022 mm in length. Occasionally they are arranged in groups (Figure 30B), but during preparation they tend to dissociate and become singles. SEM revealed the unique microstructure of the sclerites, which comprise densely packed chip-like microscleres (Figure 30C), giving the sclerite surface the appearance of cement-chip aggregates (Figure 30D).

######## Color.

Live colonies are brown with yellow polyps (Figures 5C–D).

######## Remarks.

The original description of the type by Thomson & Dean (1931: 34) is in agreement with the current findings, and indicates 10–12 pinnules compared to 12–14 noted by us. The sclerite size of 0.01 mm as given in the original description is incorrect and was later corrected by Verseveldt (1971). The latter study provides a better description of the sclerites as oblong, pear-like or angular in shape, 0.015–0.021 mm in diameter. The light microscopy used in the past clearly could not have revealed the unique surface microstructure of that species (Figure 30D).

**Figure 31.**
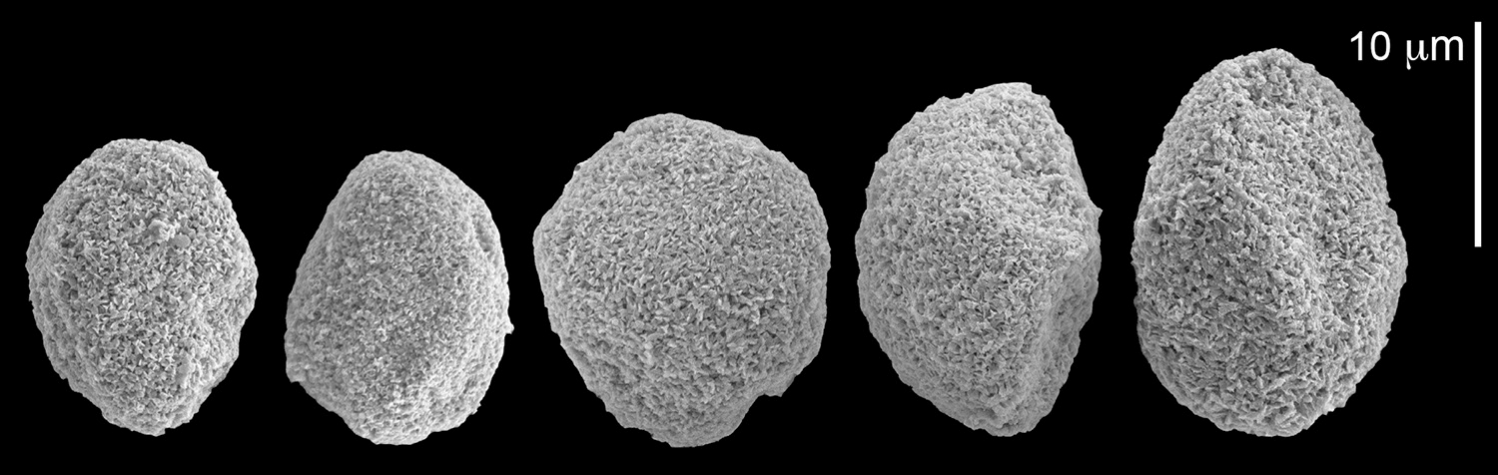
*Cespitularia turgida* Verseveldt, 1971, RMNH Coel 6607.

Examination of the type of *Cespitularia turgida* Verseveldt, 1971 (RMNH Coel 6607) revealed *Caementabunda*-type sclerites (Figure 31). In the original description Verseveldt (1971: 62) presented a comparison between the type of *C. simplex* and his new species and noted the number of pinnules in the single row of both species being 10–12 in the latter *vs.* 5–6 in the former. The current examination of the type of *C. turgida* has confirmed the original morphological findings, while we also present here for the first time images of its sclerites.

**Figure 32.**
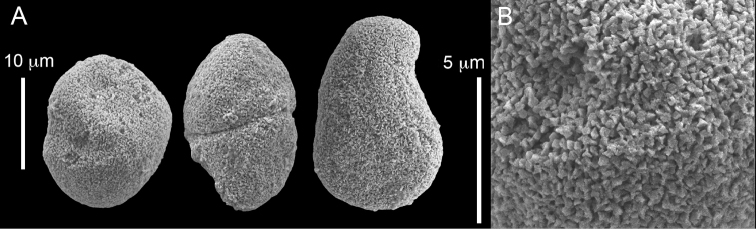
*Caementabunda simplex* (Thomson & Dean, 1931), BMNH 1982.11.17. **A** spheroid sclerites **B** densely packed chips-like microscleres of spheroid’s surface.

**Figure 33.**
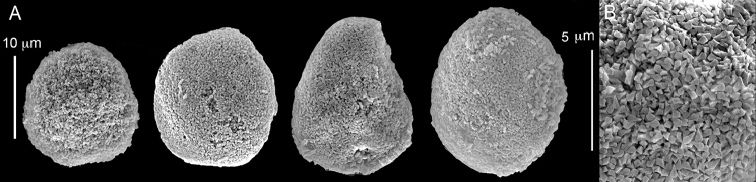
*Caementabunda simplex* (Thomson & Dean, 1931), BMNH 1982.11.18. **A** spheroid sclerites **B** densely packed chips-like microscleres of spheroid’s surface.

**Figure 34.**
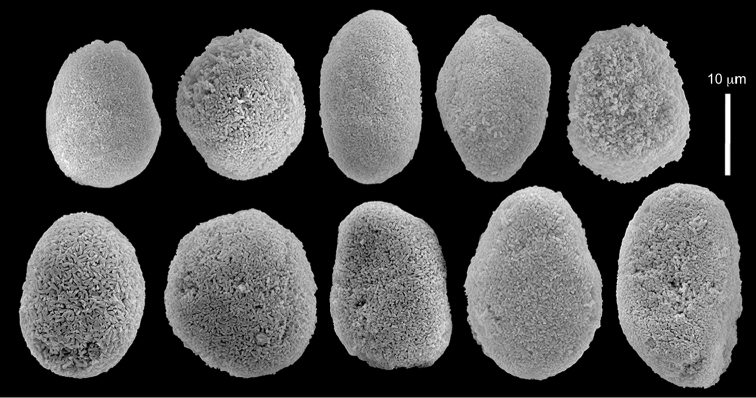
*Caementabunda simplex* (Thomson & Dean, 1931), ZMTAU Co 35757. Spheroid sclerites.

**Figure 35.**
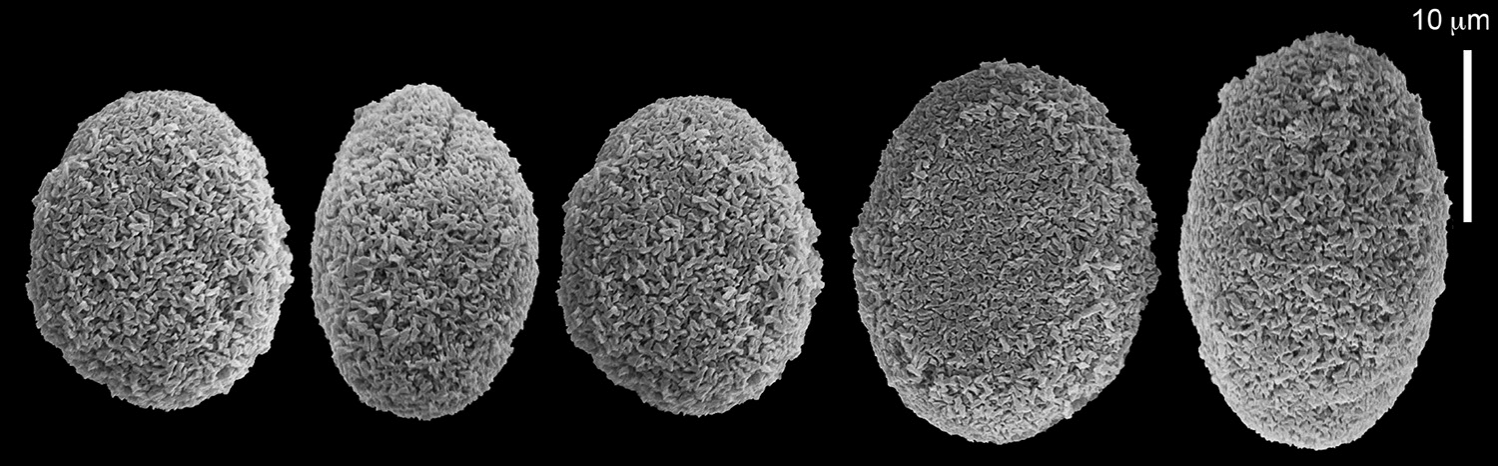
*Caementabunda simplex* (Thomson & Dean, 1931), ZMTAU Co 36122. Spheroid sclerites.

Dr. Zena Dinesen (Department of Agriculture, Fisheries and Forestry, Queensland) provided us with an unpublished taxonomic manuscript dealing with some Xeniidae of Flinders Reefs, Great Barrier Reef. Under the collection numbers BMNH 1982.11.17 and 1982.11.18 there are colonies labeled as paratypes of *Efflatounaria flindensis* Dinesen. Recently Dr. Dinesen confirmed that these two colonies are provisional paratypes of unpublished species presented in her manuscript. Our examination of the colonies revealed *Caementabunda*-type sclerites (BMNH 1982.11.17: figure 32, 1982.11.17: figure 33). In addition, it confirmed the unpublished morphological description of the material which states that the pinnules: “Mostly very contracted, difficult to measure, in one row on each side of the tentacle with 5–12 (6–9) pinnules per row”. Hence, the pinnule number corresponds to the original types of both *C. simplex* and of *C. turgida*. Similarly, examination of ZMTAU Co 35757 from Taiwan revealed *Caementabunda*-type sclerites (Figure 34) and 10–12 pinnules in a row, and ZMTAU Co 36127 and Co 36122 from Madagascar both had *Caementabunda*-type sclerites (Co 36122: figure 35) and 7–11 pinnules, thus falling within the range stated above. Based on these findings, it is concluded here that pinnule count is not diagnostic for species delineation in the newly-described genus *Caementabunda*. Similarly, it is concluded that *Cespitularia turgida* is a junior synonym of *Caementabunda simplex* and thus that both should be accommodated within this new genus.

######## Other material.

**Figure 36.**
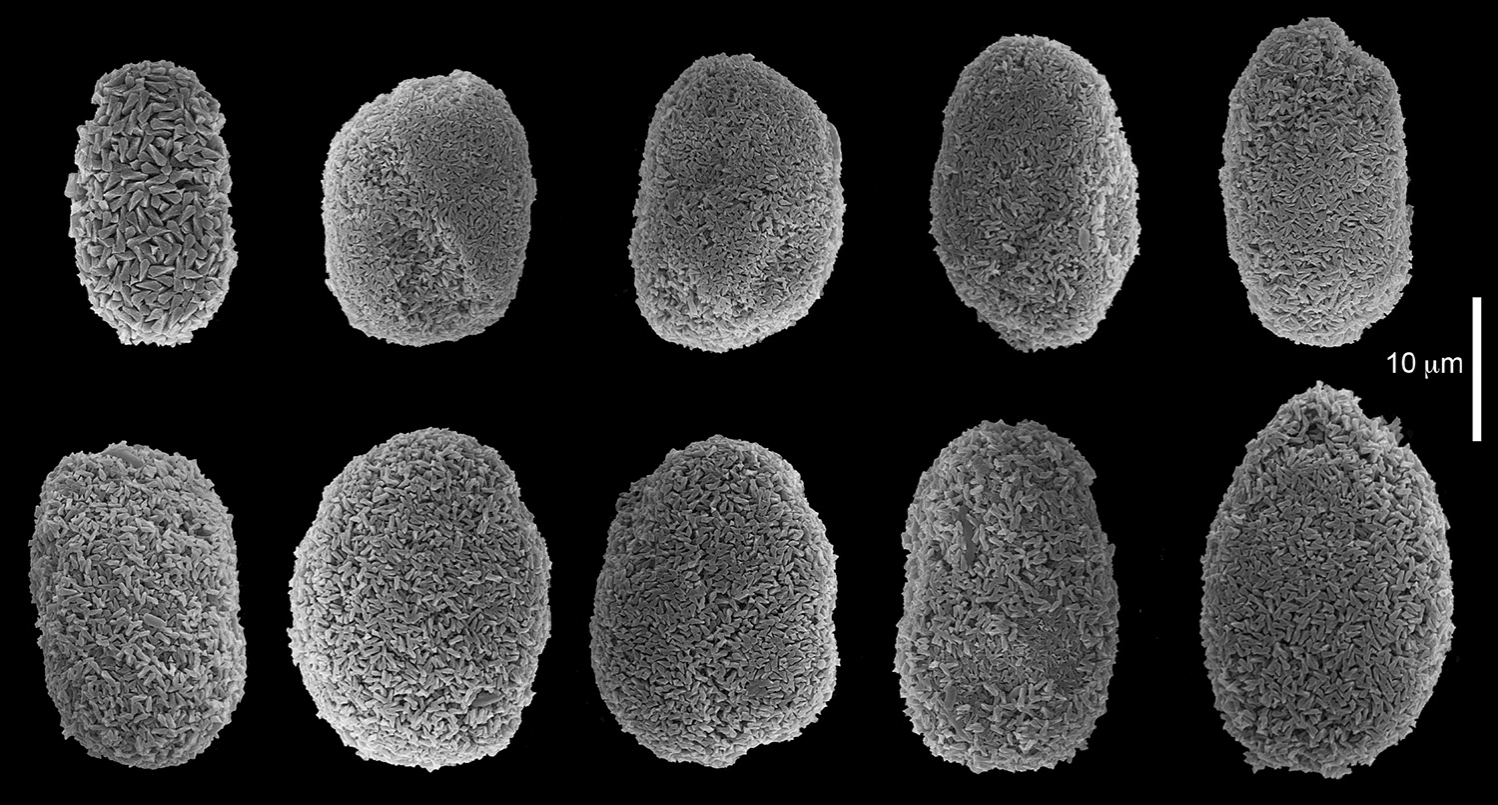
*Caementabunda simplex* (Thomson & Dean, 1931), ZMTAU Co 31642. Spheroid sclerites.

**Figure 37.**
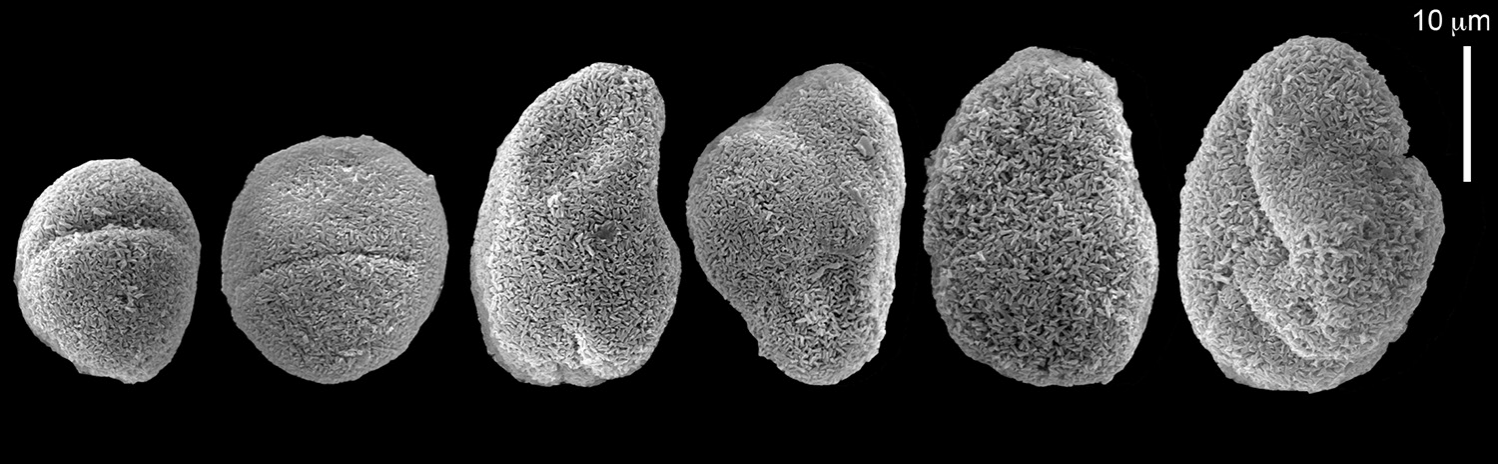
*Caementabunda simplex* (Thomson & Dean, 1931), USNM 60793. Spheroid sclerites.

All other material (see above) features the same sclerites described above for the syntype (Figure 30). Macfadyen (1936: 27) described in a colony from the Great Barrier Reef Expedition numerous minute discs about 0.010 mm in diameter, finely sculptured. The current examination of that colony (BMNH 1934.3.28.8) revealed *Caementabunda*-type sclerites. Likewise, RMNH Coel 38673 from Seychelles (see Janes 2008) and ZMTAU Co 31642 (Figure 36) feature this type of sclerite, as do USNM 60793 and 60794 collected in the Philippines (USNM 60793: Figure 37). Based on the current findings all of these colonies were assigned to the new genus.

######## Distribution.

Green Island, Taiwan; Philippines; Great Barrier Reef; Sulawesi; Madagascar; Seychelles.

######## Molecular phylogenetic results.

Sequences of *mtMutS* (582 bp), *igr1+COI* (767 bp) and *28S rDNA* (755 bp) were obtained from 46 individuals of *Conglomeratusclera* and nine individuals of *Caementabunda* from three different geographical locations: Madagascar; Green Is., Taiwan; Yonaguni Is., Japan (GenBank accession nos. MH071812–MH071969). All phylogenetic analyses of individual gene regions as well as the concatenated alignment (2104 bp) recovered trees in which specimens of *Conglomeratusclera* and *Caementabunda* formed two separate, well-supported clades (Figure 6). The average pairwise genetic distance (K2p) among individuals belonging to the two different clades was 3.6%, a value comparable to or higher than that observed among most other genera of xeniids (Figure 6).

All individuals of *Conglomeratusclera* shared identical sequences at *mtMutS* and *COI*, with just two exceptions: a single individual from Taiwan (ZMTAU Co35731) that differed by 0.2% at *mtMutS*; and one from Madagascar (ZMTAU Co36055) that differed by 0.4% at *COI*. Variation at the *28S rDNA* locus ranged from 0–1.5%. Although a group of nine *Conglomeratusclera* colonies from Taiwan shared a *28S* genotype that differed from all others by three nucleotide substitutions (0.4%), there was no significant bootstrap or *a posteriori* support for them as a separate clade, and no obvious morphological differences to suggest that they might represent a different species.

All *Caementabunda* specimens also shared identical *mtMutS* and *COI* sequences, with the exception of a single individual (ZMTAU Co 36076) that differed by 0.1% at *COI*. At *28S* rDNA pairwise genetic distances (K2p) among individuals ranged from 0–0.8%, and a group of three specimens from Madagascar (ZMTAU Co 36065, Co 36076, Co 36122) differed from all others by three nucleotide substitutions. There was, however, no significant support for this clade, and no apparent morphological differences between these individuals and others of *C. simplex*.

## Conclusions

Morphological and molecular phylogenetic analyses support the reassignment of the former species *Cespitularia coerulea* and *C. simplex* into two separate genera; *Conglomeratusclera* n. gen. and *Caementabunda* n. gen., respectively. They are distinguished by differences in sclerite microstructure as well as genetic distances comparable to those among other well-defined genera of the family Xeniidae. In addition, the findings justify synonymy of *C. taeniata* and *C. turgida* with each of these two new genera, respectively. We are at present only able to distinguish a single species in each of the new genera, based on both morphology and genetics. It should be noted that the status of *C. robusta* as a second species of *Conglomeratusclera* remains to be verified genetically. A recent study of the xeniid genus *Ovabunda* found a lack of congruence between the morphological characters traditionally used to diagnose species, in particular the number of rows of pinnules and pinnules per row, and genetic evidence of species boundaries (McFadden et al. 2017). In that case, evidence from multiple segregating nuclear markers was necessary to delineate species that shared identical or very similar mitochondrial haplotypes. Therefore, it is possible that data from additional genetic markers might detect further differences among those individuals with variant 28S rDNA genotypes that we have assigned here to *Conglomeratusclera coerulea* and *Caementabunda simplex*. As currently circumscribed, both of these new genera and in particular the respective species occur over a wide geographic range from the south-western Indian Ocean (Madagascar) to Japan.

## Supplementary Material

XML Treatment for Conglomeratusclera

XML Treatment for Conglomeratusclera coerulea

XML Treatment for Conglomeratusclera robusta

XML Treatment for Caementabunda

XML Treatment for Caementabunda simplex

## References

[B1] Aharonovich D Benayahu Y (2011) Microstructure of octocoral sclerites for diagnosis of taxonomic features. Marine Biodiversity 42: 173–177. https://doi.org/10.1007/s12526-011-0102-3

[B2] Alderslade P (2001) Six new genera and six new species of soft corals, and some proposed familial and subfamilial changes within the Alcyonacea (Coelenterata: Octocorallia). Bulletin of the Biological Society of Washington 10: 15–65.

[B3] Benayahu Y (1990) Xeniidae (Cnidaria: Octocorallia) from the Red Sea with description of a new species. Zoologische Mededelingen Leiden 64: 113–120.

[B4] Benayahu Y (2010) A new genus of a soft coral of the family Xeniidae (Cnidaria: Octocorallia) from Japan. Galaxea, Journal of Coral Reef Studies 12: 53–64. https://doi.org/10.3755/galaxea.12.53

[B5] Benayahu Y Jong MS Perkol-Finkel S Dai CF (2004) Soft corals (Octocorallia: Alcyonacea) from southern Taiwan: II. Species diversity and distributional patterns. Zoological Studies 43: 548–560.

[B6] Brundin JAZ (1896) Alcyonarien aus der Sammlung des zoologischen Museums in Upsala. Bihang till Kongl. Svenska Vetenskaps-Akademiens Handlingar XXII, Afd. IV, N 3: 1–22.

[B7] Cordeiro R Ofwegen L van Williams G (2018) World List of Octocorallia *Cespitularia* Milne Edwards & Haime, 1850. Accessed through World Register of Marine Species. http://marinespecies.org/aphia.php?p=taxdetails&id=205452 [2018–02–21]

[B8] Darriba D Taboada GL Doallo Posada D (2012) jModelTest 2: more models, new heuristics and parallel computing. Nature Methods 9: 772. https://doi.org/10.1038/nmeth.2109 10.1038/nmeth.2109 PMC4594756 22847109

[B9] Fabricius K Alderslade P (2001) Soft Corals and Sea Fans: A Comprehensive Guide to the Tropical Shallow-Water Genera of the Central-West Pacific, the Indian Ocean and the Red Sea. Townsville, Australian Institute of Marine Science, 264 pp.

[B10] Folmer O Black M Hoeh W Lutz R Vrijenhoek R (1994) DNA primers for amplification of mitochondrial cytochrome c oxidase subunit I from diverse metazoan invertebrates. Molecular Marine Biology & Biotechnology 3: 294–299. 7881515

[B11] Gohar HAF (1938) On a new species of *Cespitularia* and two invalid species of *Xenia* and *Clavularia*. Journal of Zoology, B 108(3): 483–487. https://doi.org/10.1111/j.1469-7998.1938.tb08526.x

[B12] Halàsz A McFadden CS Aharonovich D Toonen R Benayahu Y (2014) A revision of the octocoral genus *Ovabunda* Alderslade, 2001 (Anthozoa, Octocorallia, Xeniidae). Zookeys 373: 1–41. https://doi.org/10.3897/zookeys.373.6511. 10.3897/zookeys.373.6511 PMC3909805 24493958

[B13] Halàsz A Reynolds AM McFadden CS Toonen RJ Benayahu Y (2015) Could polyp pulsation be the key to species boundaries in the genus *Ovabunda* (Octocorallia: Alcyonacea: Xeniidae)? Hydrobiologia 759: 95–107. https://doi.org/10.1007/s10750-014-2106-z

[B14] Haverkort-Yeh RD McFadden CS Benayahu Y Berumen M Halàsz A Toonen RJ (2013) A taxonomic survey of Saudi Arabian Red Sea octocorals (Cnidaria: Alcyonacea). Marine Biodiversity 43: 279–291. https://doi.org/10.1007/s12526-013-0157-4

[B15] Hickson SJ (1931) The alcyonarian family Xeniidae, with a revision of the genera and species. Great Barrier Reef Expedition 1928–29, Scientific Reports, British Museum 4(5): 137–179.

[B16] Janes MP (2008) A study of the Xeniidae (Octocorallia, Alcyonacea) collected on the “Tyro” expedition to the Seychelles with a description of a new genus and species. Zoologische Mededelingen Leiden 82(49): 599–626.

[B17] Janes MP (2013) Distribution and diversity of the soft coral family Xeniidae (Coelenterata: Octocorallia) in Lembeh Strait, Indonesia. Galaxea, Journal of Coral Reef Studies (Special Issue): 195–200. https://doi.org/10.3755/galaxea.15.195

[B18] Katoh K Kuma K Toh H Miyata T (2005) MAFFT version 5: improvement in accuracy of multiple sequence alignment. Nucleic Acids Research 33: 551–513. https://doi.org/10.1093/nar/gki198 10.1093/nar/gki198 PMC548345 15661851

[B19] Kükenthal W (1902) Versuch einer Revision der Alcyonarien: I. Die Familie der Xeniiden. Zoologisches Jahrbüch, Abteilung für systematic, Geographie und Biologie der Tiere 15: 635–662. https://doi.org/10.5962/bhl.part.19040

[B20] Lamarck MC (1816) Les Caracteres Generaux et Particuliers de ces Animaux, leur Distribution, leur Classes, leurs Familles, leurs Genres, et la Citation des Principales Especes qui s’y Rapportent. Histoire Naturelle des Animaux sans Vertebres (2): 388–421.

[B21] Macfadyen LMI (1936) Alcyonaria (Stolonifera, Alcyonacea, Telestacea and Gorgonacea). Great Barrier Reef Expedition 1928–29, Scientific Reports, British Museum 5(2): 19–71.

[B22] Malyutin AN (1992) Octocorallia from the Seychelles Islands with some ecological observations. Atoll Research Bulletin 367: 1–4. https://doi.org/10.5479/si.00775630.367.1

[B23] May W (1898) Die von Dr. Stuhlmann im Jahre 1889 gesammelten ostafrikanischen Alcyonaceen des Hamburger Museums. Mitteilungen des naturhistorischen Museums Hamburg 15 (Doctoral dissertation), Supplement 2: 3–38.

[B24] May W (1899) Beiträge zur Systematik und Chorologie der Alcyonaceen. Jenaische Zeitschrift Naturwissenschaften 33 (Neue Folge 26): 1–180.

[B25] McFadden CS Tullis ID Hutchinson MB Winner K Sohm JA (2004) Variation in coding (NADH dehydrogenase subunits 2, 3 and 6) and non-coding intergenic spacer regions of the mitochondrial genome in Octocorallia (Cnidaria: Anthozoa). Marine Biotechnology 6: 516–526. https://doi.org/10.1007/s10126-002-0102-1 15723254 10.1007/s10126-002-0102-1

[B26] McFadden CS France SC Sánchez JA Alderslade P (2006) A molecular phylogenetic analysis of the Octocorallia (Coelenterata: Anthozoa) based on mitochondrial protein-coding sequences. Molecular Phylogenetics and Evolution 41: 513–527. https://doi.org/10.1016/j.ympev.2006.06.010 16876445 10.1016/j.ympev.2006.06.010

[B27] McFadden CS Benayahu Y Pante E Thoma JN Nevarez PA France SC (2011) Limitations of mitochondrial gene barcoding in Octocorallia. Molecular Ecology Resources 11: 19–31. https://doi.org/10.1111/j.1755-0998.2010.02875.x 21429097 10.1111/j.1755-0998.2010.02875.x

[B28] McFadden CS Ofwegen LP van (2013) A second, cryptic species of the soft coral genus *Incrustatus* (Anthozoa: Octocorallia: Clavulariidae) from Tierra del Fuego, Argentina revealed by DNA barcoding. Helgoland Marine Research 67: 137–147. https://doi.org/10.1007/s10152-012-0310-7

[B29] McFadden CS Reynolds AM Janes MP (2014) DNA barcoding of xeniid soft corals (Octocorallia: Alcyonacea: Xeniidae) from Indonesia: species richness and phylogenetic relationships. Systematics & Biodiversity 12: 247–257. https://doi.org/10.1080/14772000.2014.902866

[B30] McFadden CS Haverkort-Yeh R Reynolds AM Halàsz A Quattrini AM Forsman Z Benayahu Y Toonen RJ (2017) Species boundaries in the absence of morphological, ecological or geographical differentiation in the Red Sea octocoral genus *Ovabunda* (Alcyonacea: Xeniidae). Molecular Phylogenetics & Evolution 112: 174–184. https://doi.org/10.1016/j.ympev.2017.04.025 28467886 10.1016/j.ympev.2017.04.025

[B31] Milne Edwards H Haime J (1850) A monograph of the British fossil corals – Part I: Introduction, corals from the Tertiary and Cretaceous formation. Palaeontographical Society, London, 71 pp.

[B32] Quoy JRC Gaimard P (1833) Zoophytes. In: Voyage de découvertes de l’Astrolabe executé par ordre du Roi, pendant les années 1826–1827–1828–1829, sous le commandement de M. J. Dumont d’Urville Zoologie 4: 1–390.

[B33] Reinicke GB (1997) Xeniidae (Coelenterata: Octocorallia) of the Red Sea with descriptions of six new species of *Xenia*. Fauna of Saudi Arabia 16: 5–62.

[B34] Ronquist F Teslenko M van der Mark P Ayres D Darling A Höhna S Larget B Liu L Suchard MA Huelsenbeck JP (2012) MrBayes 3.2: Efficient Bayesian phylogenetic inference and model choice across a large model space. Systematic Biology 61: 539–542. https://doi.org/10.1093/sysbio/sys029 22357727 10.1093/sysbio/sys029 PMC3329765

[B35] Roxas HA (1933) Philippine Alcyonaria. The families Cornulariidae and Xeniidae. The Philippine Journal of Science 50: 49–108.

[B36] Ruiz-Allais JP Amaro ME McFadden CS Halàsz A Benayahu Y (2014) The first incidence of an alien soft coral of the family Xeniidae in the Caribbean, an invasion in eastern Venezuelan coral communities. Coral Reefs 33: 287–287. https://doi.org/10.1007/s00338-013-1122-1

[B37] Sánchez JA McFadden CS France SC Lasker HR (2003) Phylogenetic analyses of shallow-water Caribbean octocorals. Marine Biology 142: 975–987. https://doi.org/10.1007/s00227-003-1018-7

[B38] Tamura K Peterson D Peterson N Stecher G Nei M Kumar S (2011) MEGA5: molecular evolutionary genetics analysis using maximum likelihood, evolutionary distance, and maximum parsimony methods. Molecular Biology and Evolution 28: 2731–2739. https://doi.org/10.1093/molbev/msr121 21546353 10.1093/molbev/msr121 PMC3203626

[B39] Thomson JA Dean LMI (1931) Alcyonacea of the Siboga Expedition. Siboga- Expedition Monograph 13d: 1–227.

[B40] Thomson JA Henderson WD (1906) The Marine Fauna of Zanzibar and British East Africa, from Collections made by Cyril Crossland, MA, B. Sc., FZS, in the Years 1901 and 1902. Alcyonaria. Journal of Zoology 76: 393–443.

[B41] Thomson JA Mackinnon DL (1910) Alcyonarians collected on the Percy Sladen Trust Expedition by Mr. J. Stanley Gardiner. Part II. The Stolonifera, Alcyonacea, Pseudaxonia, and Stelechotokea. Transactions of the Linnean Society of London 13(8): 165–211. [pls. 6–14]

[B42] Tilot V Leujak W Ormond RFG Ashworth JA Mabrouk A (2008) Monitoring of South Sinai coral reefs: influence of natural and anthropogenic factors. Aquatic Conservation: Marine and Freshwater Ecoystems 18: 1109–1126. https://doi.org/10.1002/aqc.942

[B43] Tixier-Durivault A (1966) Faune de Madagascar. XXI: Octocoralliaires. ORSTOM, Paris.

[B44] Utinomi H (1950) Some Xeniid Alcyonarians from Japan and Adjacent Localities. Publications of the Seto Marine Biological Laboratory 1(3): 7–17. https://doi.org/10.5134/174440

[B45] Utinomi H (1954) Some alcyoniid octocorals from Kii coast, middle Japan. Publications of the Seto Marine Biological Laboratory 4(1): 43–55. https://doi.org/10.5134/174502

[B46] Verseveldt J (1971) Octocorallia from North-Western Madagascar (Part II). Zoologische Verhandelingen 117: 1–73.

[B47] Wild C Naumann MS (2013) Effect of active water movement on energy and nutrient acquisition in coral reef-associated benthic organisms. Proceedings of the National Academy of Sciences of the United States of America 110: 8767–8768. https://doi.org/10.1073/pnas.1306839110 23671104 10.1073/pnas.1306839110 PMC3670397

[B48] Zwickl DJ (2006) Genetic algorithm approaches for the phylogenetic analysis of large biological sequence datasets under the maximum likelihood criterion. Ph.D. dissertation, University of Texas: Austin.

